# A system-level approach identifies HIF-2α as a critical regulator of chondrosarcoma progression

**DOI:** 10.1038/s41467-020-18817-7

**Published:** 2020-10-06

**Authors:** Hyeonkyeong Kim, Yongsik Cho, Hyeon-Seop Kim, Donghyun Kang, Donghyeon Cheon, Yi-Jun Kim, Moon Jong Chang, Kyoung Min Lee, Chong Bum Chang, Seung-Baik Kang, Hyun Guy Kang, Jin-Hong Kim

**Affiliations:** 1 Center for RNA Research, Institute for Basic Science, 08826 Seoul, South Korea; 2 Department of Biological Sciences, College of Natural Sciences, Seoul National University, 08826 Seoul, South Korea; 3 Department of Radiation Oncology, Ewha Womans University College of Medicine, 07985 Seoul, South Korea; 4 Department of Orthopaedic Surgery, Seoul National University College of Medicine, Boramae Hospital, 07061 Seoul, South Korea; 5 Department of Orthopaedic Surgery, Seoul National University Bundang Hospital, 13620 Seongnam, South Korea; 6 Orthopedic Oncology Clinic, Specific Organs Cancer Branch, Research Institute and Hospital, National Cancer Center, 10408 Goyang, South Korea; 7 Interdisciplinary Program in Bioinformatics, Seoul National University, 08826 Seoul, South Korea

**Keywords:** Targeted therapies, Sarcoma

## Abstract

Chondrosarcomas, malignant cartilaginous neoplasms, are capable of transitioning to highly aggressive, metastatic, and treatment-refractory states, resulting in significant patient mortality. Here, we aim to uncover the transcriptional program directing such tumor progression in chondrosarcomas. We conduct weighted correlation network analysis to extract a characteristic gene module underlying chondrosarcoma malignancy. Hypoxia-inducible factor-2α (HIF-2α, encoded by *EPAS1*) is identified as an upstream regulator that governs the malignancy gene module. HIF-2α is upregulated in high-grade chondrosarcoma biopsies and *EPAS1* gene amplification is associated with poor prognosis in chondrosarcoma patients. Using tumor xenograft mouse models, we demonstrate that HIF-2α confers chondrosarcomas the capacities required for tumor growth, local invasion, and metastasis. Meanwhile, pharmacological inhibition of HIF-2α, in conjunction with the chemotherapy agents, synergistically enhances chondrosarcoma cell apoptosis and abolishes malignant signatures of chondrosarcoma in mice. We expect that our insights into the pathogenesis of chondrosarcoma will provide guidelines for the development of molecular targeted therapeutics for chondrosarcoma.

## Abstract (web summary)

Chondrosarcomas are frequently aggressive, understanding the transcriptional changes associated with progression may help in developing new treatments. Here, the authors show that HIF-2α is increased in expression on progression and pharmacological inhibition of the protein together with chemotherapy is a useful strategy for controlling tumour growth in mice.

## Introduction

Chondrosarcomas are common primary neoplasms arising within the bone, constituting one-third of skeletal system cancers^1,2^. The vast majority (>85%) of chondrosarcomas are central chondrosarcomas that arise from an intramedullary location^3^, surrounded by thick layers of bone matrix and rarely exhibiting metastatic cell survival and outgrowth^1,2^. These types of low-grade chondrosarcomas remain dormant without noticeable symptoms. In contrast, high-grade chondrosarcomas contribute to significant patient morbidity as they are often highly metastatic^1^. Chondrosarcomas frequently recur after excision surgery, and recurrent tumors tend to attain a higher malignancy status than the original neoplasm^2,3^. Moreover, chondrosarcoma can further transition into dedifferentiated chondrosarcoma^4^, a subtype with the highest mortality rate^5^.

High-grade chondrosarcomas are typically treatment-refractory as they are resistant to conventional chemotherapy and radiotherapy^1,2^. Moreover, unlike other mesenchymal malignancies, such as osteosarcoma and Ewing sarcoma, for which several effective targeted therapies have been devised^6,7^, limited clinical evidence exists to clearly indicate an effective therapy for high-grade chondrosarcoma, nor has the survival rate been significantly improved over the past several decades^8^.

HIF-2α is a member of the basic helix-loop-helix/PAS transcription factor family. Its amino acid sequence shares 48% homology with that of HIF-1α (encoded by *HIF1A*)^9^. HIF-2α, like HIF-1α, is regulated via oxygen-dependent degradation and modulates the hypoxic response^10^. However, emerging evidence suggests that HIF-2α plays distinct roles in a range of developmental and pathogenic processes^11,12^.

The most frequent driver mutations associated with chondrosarcoma occur in the isocitrate dehydrogenase (*IDH*) genes. In particular, mutations are exclusively present in catalytic arginine residues such as R132 of *IDH1*, and R140 or R172 of *IDH2*^13,14^. The mutant IDHs acquire a neomorphic activity that reduces α-ketoglutarate (α-KG) to D-2-hydroxyglutarate (D2HG), which is a known oncometabolite^15,16^. D2HG competes with α-KG for binding to the active sites of α-KG-dependent enzymes, such as prolyl hydroxylases, presumably resulting in a pseudohypoxic state^17^. Aside from *IDH*, mutations in *COL2A1*^18^, *NRAS*^19^, and *YEATS2*^20^ have also been documented, providing insights into the genetic basis of chondrosarcoma development. Meanwhile, chondrosarcomas are notoriously heterogeneous with respect to their genomic and histopathological features. In fact, the heterogeneity of the chondrosarcoma genome presents a major obstacle for the development of effective therapeutic strategies for this disease^4,21^.

Here, we conduct unsupervised gene co-expression network analyses using transcriptomes of patients with chondrosarcoma and extract a characteristic transcription network underlying chondrosarcoma malignancy. By implementing a system-level upstream analysis of this gene network, we identify HIF-2α as a key factor governing chondrosarcoma malignancy. We unravel the functional roles of HIF-2α in promoting tumor growth and metastasis of chondrosarcomas in the context of their unique microenvironments. Furthermore, our findings present an opportunity for the development of effective combination therapy for treating chondrosarcoma.

## Results

### HIF-2α is a predicted transcriptional regulator of malignancy in chondrosarcoma patients

**Fig. 1:**
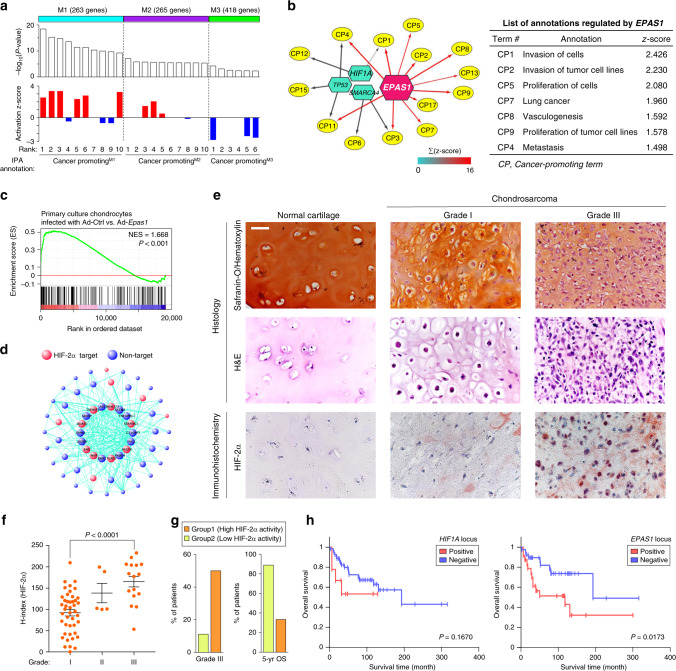
HIF-2α is an upstream regulator of gene module governing chondrosarcoma malignancy. **a** Three moderately sized modules identified by WGCNA using transcriptome data from chondrosarcoma patients (GSE12475). Cancer-promoting (CP) annotations, significantly associated with each module, were analyzed using IPA. For each module, top ten CP annotations were arranged in the order of –log_10_(*P*-value). –log_10_(*P*-value) > 2 was used as the cut-off value. A right-tailed Fisher’s exact test was used to determine the statistical significance. **b** The predicted upstream regulators of the M1 module were color-coded according to the sum of activation z-scores of linked nodes. Edges from *EPAS1* are highlighted in red and their thickness indicates significance according to *P*-value against each annotation (left panel). Representative CP annotations, regulated by *EPAS1*, were arranged in the order of activation z-score (right panel). A right-tailed Fisher’s exact test was used to determine the statistical significance. **c** GSEA was performed with the M1 module gene set using transcriptome data obtained from chondrocytes infected with adenovirus (Ad)-*Epas1* compared to those infected with Ad-Control (Ctrl) (GSE73659). Normalized enrichment score (NES) and nominal *P*-value are indicated. **d** Network connections of M1 module genes visualized by VisANT. The hub genes are located toward the center of the network. **e** Representative histological and IHC images in normal human cartilage and chondrosarcoma biopsies. Scale bar: 50 μm. **f** Quantitation of HIF-2α expression in chondrosarcomas with different histological grades: grade I (*n* = 44), grade II (*n* = 5) and grade III (*n* = 16). Significance was calculated by one-way ANOVA. Data represent mean ± SEM. **g** Patients were grouped by conducting *k*-means clustering based on collective expression profiles of canonical HIF-2α target genes (Supplementary Fig. 1c). Clinical and prognostic features of patients in Group1 and Group2 are presented by their percentage in each group. 5-year OS; 5-year overall survival. **h** Kaplan–Meier plot of overall survival of the patients stratified by amplification status of *HIF1A* (left panel; positive: *n* = 9; negative: *n* = 55) and *EPAS1* (right panel; positive: *n* = 24; negative: *n* = 40) loci. Significance was calculated with the two-sided log-rank test.

To extract a characteristic gene expression pattern underlying chondrosarcoma malignancy, we conducted weighted gene co-expression network analysis (WGCNA)^22^ based on the transcriptome dataset of chondrosarcoma patients^23^. Six distinct gene modules, each consisting of highly correlated module membership genes, were identified (Supplementary Data 1). Each module was then analyzed by calculating enrichment *P*-values and activation z-scores for all cancer-promoting annotations obtained from Ingenuity Pathway Analysis (IPA)^24^. The three large modules (L1: 1,451 genes, L2: 3,459 genes, and L3: 3,343 genes) did not exhibit distinct enrichment patterns for cancer-promoting annotations (Supplementary Fig. 1a and Supplementary Table 1). We then focused on the remaining three moderately sized modules (M1: 263 genes, M2: 265 genes, and M3: 418 genes) that activation of which may be governed by a single transcriptional unit in human chondrosarcoma (Fig. 1a and Supplementary Table 1). Notably, the M1 module revealed significant enrichment patterns for cancer-promoting annotations such as *growth of tumor*, metastasis, and advanced malignant tumor, with most being in the highly activated state upon module activation. In contrast, the M2 and M3 modules had relatively lower extents of overlap with cancer-promoting terms (Fig. 1a). We, therefore, defined the M1 module as a functional gene set, the expression of which may dictate malignant characteristics of chondrosarcoma.

We then performed IPA upstream regulator analysis to predict the transcriptional regulator governing the expression of M1 module genes. HIF-2α was predicted to be the most potent transcription factor activating this module (Fig. 1b and Supplementary Table 2). Indeed, our gene set enrichment analysis (GSEA)^25^ indicated that overexpression of HIF-2α in chondrocytes elicits overall upregulation of M1 module genes at the transcriptome level (Fig. 1c). Based on the WGCNA analysis^22^, hub genes (i.e., those with high connectivity) within the M1 module gene network appear to have a strong tendency to be the transcriptional targets of HIF-2α, further supporting HIF-2α-driven collective activation of M1 module genes (Fig. 1d and Supplementary Table 3). Immunohistochemistry (IHC) analysis of normal cartilage, osteochondroma, enchondroma, and chondrosarcoma tissues indicated an overall upregulation of HIF-2α protein in chondrosarcoma biopsies (Supplementary Fig. 1b). Notably, using a panel of chondrosarcoma biopsies, we observed that high expression of HIF-2α was more significantly associated with grade III than grade I chondrosarcoma (Fig. 1e, f and Supplementary Data 2). Therefore, we examined a possible correlation between the predicted transcriptional activity of HIF-2α and clinical outcomes in patients. To this end, we classified patients with chondrosarcoma into two groups, according to the transcription profiles of HIF-2α target genes such that group 1 correlated with a higher activation status of HIF-2α compared to group 2 (Supplementary Fig. 1c and Supplementary Table 4). We then cross-checked the clinical data of the patients in each group for correlation with the predicted HIF-2α activity. Results show that patients in group 1 tended to be more frequently associated with grade III chondrosarcoma and to have a poorer prognosis (Fig. 1g and Supplementary Table 5).

Finally, we investigated whether potential associations exist between gene amplification at the loci of *HIF* family genes, *HIF1A* or *EPAS1*, and various aspects of chondrosarcoma malignancy, including patient prognosis. Based on the observation that various degrees of genomic instability were present in the genomic profiles of 67 patients (Supplementary Fig. 1d)^23^, we analyzed copy number alterations (CNAs) in *HIF1A* and *EPAS1* loci using the Gain and Loss Analysis of DNA (GLAD) segmentation method (Supplementary Fig. 1e)^26^. No significant differences were observed in the overall survival rates or disease-free survival rates between patients with *HIF1A* gene amplification (positive) and without (negative) (Fig. 1h and Supplementary Fig. 1 f). In contrast, amplification of the *EPAS1* gene was significantly associated with decreased overall survival rates (*P* < 0.05). Chondrosarcoma patients carrying an amplified *EPAS1* gene also tended to exhibit reduced disease-free survival compared to those without *EPAS1* amplification (*P* = 0.0559; Fig. 1h and Supplementary Fig. 1f). We then examined the relationship between the presence of *HIF1A* or *EPAS1* gene amplification and the occurrence of dedifferentiation, recurrence, or metastasis in chondrosarcoma patients. Amplification of the *HIF1A* gene did not correlate with an increased incidence of any of these features (Supplementary Table 6). In contrast, patients carrying an amplified *EPAS1* gene tended to exhibit increased dedifferentiation (*P* < 0.05) and recurrence (*P* = 0.062; Supplementary Table 6).

### HIF-2α confers a selective advantage for cancer progression in murine xenograft models

**Fig. 2:**
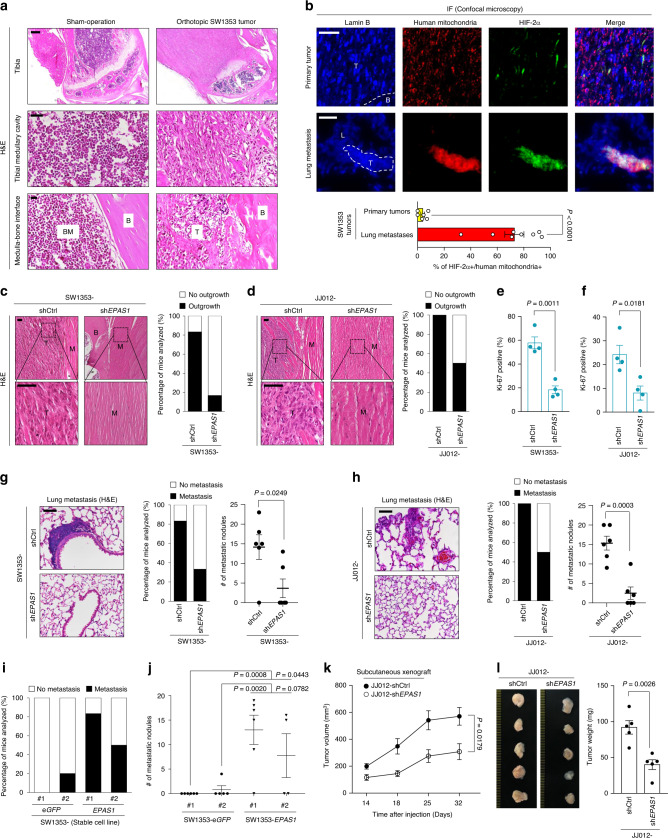
HIF-2α promotes tumor growth and metastatic propensity of chondrosarcoma in xenograft animal models. **a** Primary chondrosarcoma tumors formed in tibial intramedullary canal following orthotopic SW1353 xenograft. Images represent one of five experiments, with similar results obtained. BM, bone marrow; B, bone; T, tumor. Scale bars: 300 μm (top panel), 25 μm (middle and bottom panels). **b** IF images in primary and pulmonary metastatic tumors. T, tumor; B, bone; L, lung (upper panel). The percentage of HIF-2α positive cells among human mitochondria-positive cells (*n* = 6, primary tumors; 8, lung metastases, lower panel). Scale bars: 25 μm. **c**, **d** H&E images illustrating the extent of local invasion of **c** SW1353 and **d** JJ012 cells harboring indicated shRNAs into the muscle tissue surrounding the tibia (left panel). Percentage of mice bearing extraosseous outgrowths that originated from the intramedullary region and invaded into the surrounding muscle (*n* = 6, SW1353; 6, JJ012; right panel). M, muscle; T, tumor; B, bone. Scale bars: 50 μm. **e**, **f** Quantitation of Ki-67 positivity in primary tumors established with **e** SW1353 (*n* = 4) or **f** JJ012 (*n* = 4) cells harboring indicated shRNAs. **g**, **h** Metastatic growth of **g** SW1353 (*n* = 6) and **h** JJ012 (*n* = 6) cells harboring indicated shRNAs (left panel). The percentage of mice bearing lung metastases (middle panel) and the number of metastatic foci (right panel). Scale bars: 50 μm. **i**, **j** Pulmonary metastasis of orthotopically transplanted SW1353 cells overexpressing e*GFP* or *EPAS1* (*n* = 6, e*GFP*#1; 5, e*GFP*#2; 6, *EPAS1*#1; 4, *EPAS1*#2). **i** The percentage of mice bearing lung metastases and **j** the number of metastatic foci in the indicated groups. **k** Growth of JJ012 tumor harboring control or *EPAS1* shRNAs in the subcutaneous xenograft model (*n* = 5). The tumor volume at the indicated days after transplantation. **l** Gross images (left panel) and the weight (*n* = 5, right panel) of excised chondrosarcoma tumors at the end of the experiment. Data represent mean ± SEM. *P*-values are from two-tailed *t* test (**b**, **e**–**h**, **l**), one-way ANOVA (**j**), or two-way ANOVA (**k**).

The orthotopic mouse model of chondrosarcoma was established with the human chondrosarcoma cell lines SW1353 and JJ012^27,28^. Chondrosarcoma cells implanted within the tibial intramedullary canal developed into tumors (Fig. 2a and Supplementary Fig. 2a). These xenografted tumor cells covered the whole tibial medullary cavity while the bone marrow and endosteum were displaced by chondrosarcoma cells. Extraosseous tumor outgrowth was observed with the absorption of the tibial bone and invasion of tumor cells into the surrounding muscle tissue (Fig. 2a). A considerable heterogeneity in HIF-2α expression within these primary SW1353 tumors was observed such that a fraction of the chondrosarcoma cells exhibited distinct HIF-2α positivity (Fig. 2b and Supplementary Fig. 2b). We also noted that the transplanted chondrosarcoma cells underwent pulmonary metastasis. Interestingly, secondary SW1353 tumors in the lung were frequently determined to be HIF-2α-positive (Fig. 2b and Supplementary Fig. 2b). In keeping with this, when HIF-2α and human mitochondria were detected by multicolor immunofluorescence (IF), the percentage of double positivity among primary and metastatic SW1353 tumors was 4.6 and 70.2%, respectively (Fig. 2b). Thus, we hypothesized that HIF-2α expression may confer chondrosarcoma cells a selective advantage for escaping their primary site and becoming metastatic. To test this hypothesis, we implanted SW1353 or JJ012 cells stably transduced with sh*EPAS1* or control shRNA (Supplementary Fig. 2c–f), into the tibia of athymic mice. Knockdown of HIF-2α not only reduced proliferation of implanted chondrosarcoma cells, but also effectively reduced the occurrence of extraosseous outgrowth and pulmonary metastases (Fig. 2c–h and Supplementary Fig. 2 g, h). Next, we examined how overexpression of HIF-2α affects chondrosarcoma progression in mice. We, therefore, constructed SW1353 cells that stably overexpressed HIF-2α or eGFP (Supplementary Fig. 2i). Notably, a subset of SW1353-*EPAS1* stable cell lines spontaneously formed sarcospheres even in an adherent culture system (Supplementary Fig. 3a). Extensive secondary tumor formation was observed 7 weeks after xenograft transplantation of HIF-2α-overexpressing SW1353 cells (Fig. 2i, j and Supplementary Fig. 3b).

We further examined the role of HIF-2α in chondrosarcoma tumor growth using an alternative tumor xenograft model. Subcutaneous injection of JJ012 cells resulted in reliable tumor growth in nude mice. The stable transduction of JJ012 cells with sh*EPAS1* markedly inhibited the growth of chondrosarcoma tumors with smaller size and weight compared with the control counterparts (Fig. 2k, l).

### HIF-2α regulates differential downstream pathways distinct from HIF-1α in chondrosarcoma

**Fig. 3:**
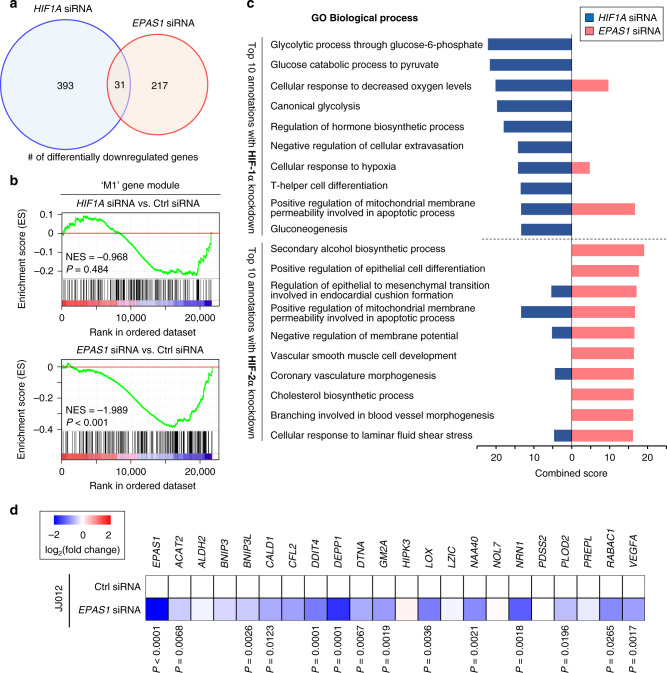
HIF-2α regulates distinct and non-redundant downstream pathways in chondrosarcoma cells compared with HIF-1α. **a** Venn diagram of differentially downregulated genes following HIF-1α or HIF-2α knockdown in SW1353 cells. **b** GSEA was performed with the M1 module gene set (Supplementary Data 1) using the transcriptome data following HIF-1α (upper panel) or HIF-2α (lower panel) knockdown. NES and nominal *P*-value are indicated. **c** Top 10-ranked Biological Process GO terms associated with differentially downregulated genes in HIF-1α or HIF-2α knockdown conditions. The combined score reflects a combination of *P*-value computed using Fisher’s exact test and z-score as explicitly described in Methods section. **d** By combining the HIF-2α ChIP-seq and our SW1353 cell transcriptome data, direct target genes of HIF-2α in chondrosarcoma cells were identified. Relative mRNA levels of these direct HIF-2α target genes were quantitated by quantitative real-time PCR (qRT-PCR) analysis in JJ012 cells. Six transcripts not detected in JJ012 were excluded from the analysis. Heatmap illustrates fold change of HIF-2α target gene abundance in JJ012 cells treated with *EPAS1* siRNA compared with those treated with Ctrl siRNA (*n* = 8). Data represent mean ± SEM. *P*-values are from two-tailed *t* test (**d**).

Although we identified a specific association between HIF-2α expression and several aspects of chondrosarcoma malignancy, there has been a general notion of redundancy between HIF-1α and HIF-2α as a common downstream effector of hypoxia. We, therefore, sought to compare downstream pathways affected by HIF-1α and HIF-2α, respectively via transcriptome analysis in SW1353 cells, with or without HIF-1α, or HIF-2α knockdown. In response to HIF-1α and HIF-2α knockdown, 424 and 248 genes were differentially downregulated, respectively (Fig. 3a and Supplementary Data 3). Interestingly, only 31 were commonly regulated by both HIFs, thus suggesting a high degree of non-redundancy for HIF-1α and HIF-2α in regard to target gene specificity in chondrosarcoma cells. In fact, GSEA indicated that the M1 module was downregulated overall by knockdown of HIF-2α but not by HIF-1α (Fig. 3b), further verifying that HIF-2α is the specific regulator of the M1 module.

Gene Ontology (GO) term enrichment analysis was performed on genes differentially downregulated upon knockdown of HIF-1α or HIF-2α. Our results demonstrated that Biological Process GO terms related to glycolysis and hypoxic responses were enriched in the HIF-1α knockdown condition (Fig. 3c), consistent with well-known functions of HIF-1α^12,29^. Interestingly, Biological Process GO terms affected by HIF-2α knockdown were not highly associated with these conventional roles of HIF family proteins in chondrosarcoma cells (Fig. 3c). Moreover, the GO analysis with Molecular Function and Cellular Component annotations also supported non-redundant, and distinct features of HIF-2α in chondrosarcoma cells compared to that of HIF-1α (Supplementary Fig. 4).

To identify target genes that are directly regulated by the binding of HIF-2α to promoter sequences, we reanalyzed publicly available HIF-2α chromatin immunoprecipitation sequencing (ChIP-Seq) datasets (GSM3417828 and GSM3417842)^30^. By combining the HIF-2α ChIP-seq and our transcriptome dataset with the knockdown of HIF-2α in SW1353 cells, we identified 27 HIF-2α direct target genes associated with HIF-2α binding peaks in the proximal regions of their transcription start sites (−5 kb to +1 kb). These HIF-2α target genes were similarly regulated in JJ012 cells (Fig. 3d).

### HIF-2α expression confers tumor-initiating and metastatic capacity to chondrosarcoma

**Fig. 4:**
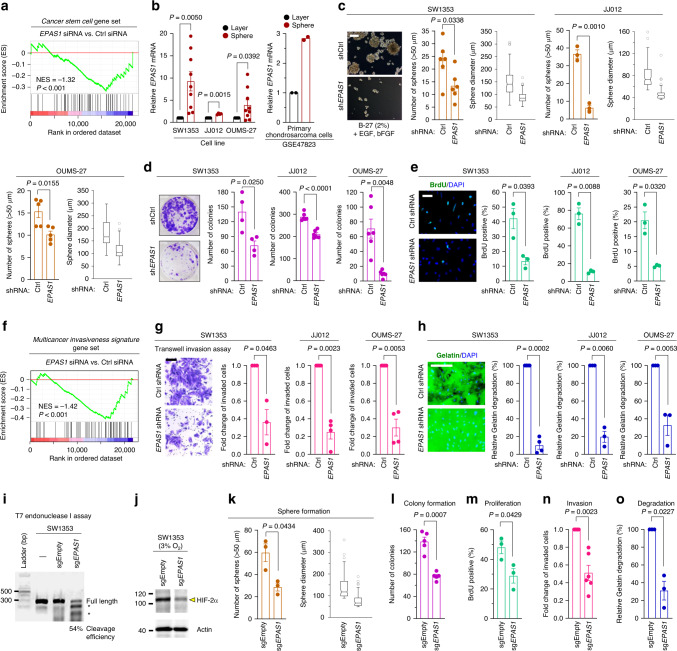
HIF-2α expression promotes tumor-initiating and invasive propensity to chondrosarcoma cells. **a** GSEA was performed for *Cancer stem cell* gene set using transcriptome data of SW1353 cells transfected with Ctrl or *EPAS1* siRNA. NES and nominal *P*-value are indicated. **b** qRT-PCR analysis of *EPAS1* transcript in SW1353 (*n* = 9), JJ012 (*n* = 4), and OUMS-27 (*n* = 9) cells, grown as a monolayer or spheres (left panel). Relative mRNA level of *EPAS1* in primary chondrosarcoma cells (GSE47823; *n* = 2, right panel). **c** The number and size of spheres were measured in SW1353 (*n* = 6), JJ012 (*n* = 3), and OUMS-27 (*n* = 5) cells transduced with the indicated lentiviruses. Scale bar: 200 μm. **d** Quantitation of colony formation assay by SW1353 (representative images, *n* = 4), JJ012 (*n* = 6), and OUMS-27 (*n* = 6) cells expressing indicated shRNAs. **e** Quantitation of the BrdU-positive cells (green) among SW1353 (representative images, *n* = 3), JJ012 (*n* = 3), and OUMS-27 (*n* = 3) cells expressing indicated shRNAs. Scale bar: 50 μm. **f** GSEA was performed for *Multicancer invasiveness signature* gene set using transcriptome data of SW1353 cells transfected with Ctrl or *EPAS1* siRNA. NES and nominal *P*-value are indicated. **g** Quantitation of invaded SW1353 (representative images, *n* = 3), JJ012 (*n* = 4), and OUMS-27 (*n* = 4) cells under indicated conditions. Scale bar: 200 μm. **h** Quantitation of relative gelatin degradation area by SW1353 (representative images, *n* = 4), JJ012 (*n* = 3), and OUMS-27 (*n* = 3) cells expressing indicated shRNAs. Scale bar: 100 μm. **i** Validation of small guide RNA (sgRNA):Cas9-mediated on-target cleavage of *EPAS1* by T7E1 cleavage assay. The hyphen above the gel image indicates wild-type gDNA of SW1353 cells not transduced with any lentivirus. Asterisks indicate cleaved PCR amplicon fragments at the expected sizes as an indicative of successful gene editing. The percentage at the bottom of the gel image indicates expected editing efficiency. **j** Immunoblot of HIF-2α protein. **k**–**o** Quantitation of **k** sphere formation assay (*n* = 3), **l** colony formation assay (*n* = 5), **m** BrdU incorporation assay (*n* = 3), **n** transwell invasion assay (*n* = 6), and **o** gelatin degradation assay (*n* = 3) in SW1353 cells transduced with lentivirus harboring Cas9 and indicated sgRNAs. Data represent mean ± SEM. *P*-values are from two-tailed *t* test (**b**–**e**, **g**, **h**, **k**–**o**). **i**, **j** Full-size agarose gel and immunoblot images are provided in Supplementary Fig. 9.

We next sought to determine whether HIF-2α affects cancer-relevant behaviors of chondrosarcoma cells. Our earlier observation that HIF-2α-overexpressing SW1353 cells form sarcospheres in an adherent culture (Supplementary Fig. 3a) suggested a possible involvement of HIF-2α in conferring cancer stemness to chondrosarcoma cells. The acquisition of cancer stemness is often associated with treatment resistance, tumor recurrence and metastasis in chondrosarcomas^31^. In fact, in response to HIF-2α knockdown, but not HIF-1α knockdown, the SW1353 transcriptome was negatively enriched in regard to the *Cancer stem cell* gene set (Fig. 4a, Supplementary Fig. 5a and Supplementary Table 7). In sphere-forming assays, HIF-2α was upregulated in sphere-forming SW1353, JJ012, and OUMS-27 cells compared with those grown as monolayers (Fig. 4b). A cross-check with the public dataset showed that the *EPAS1* mRNA level was consistently elevated in spheres of primary cultured chondrosarcoma cells compared to their monolayer counterparts (Fig. 4b). The sphere-forming potential of three chondrosarcoma cell lines was significantly impaired by HIF-2α knockdown (Fig. 4c and Supplementary Fig. 5b, c). HIF-2α knockdown also suppressed the clonogenic ability of SW1353, JJ012, and OUMS-27 chondrosarcoma cells (Fig. 4d). The effect of HIF-2α knockdown on cancer stemness was also reflected in the reduced proliferative capacity of chondrosarcoma cells both in vitro (Fig. 4e) and in vivo (Fig. 2e, f and Supplementary Fig. 2g, h).

Our results indicated that HIF-2α is implicated in promoting metastasis of chondrosarcoma cells in murine orthotopic xenograft models (Fig. 2c–j and Supplementary Fig. 3b). In chondrosarcoma, initiation of metastasis requires invasion into the surrounding matrix and migration toward the local bloodstream^32^. GSEA revealed that the *Multicancer invasiveness signature* and *Cell migration* gene sets were negatively enriched in the whole transcriptome obtained from SW1353 cells following HIF-2α, not HIF-1α, knockdown (Fig. 4f, Supplementary Fig. 5d and Supplementary Tables 8, 9). Therefore, we next investigated whether HIF-2α affects the invasive and migratory behaviors of the three chondrosarcoma cell lines, SW1353, JJ012, and OUMS-27. HIF-2α knockdown markedly suppressed cell invasiveness (Fig. 4g), whereas their migration rate was not affected (Supplementary Fig. 5e). During invasion of chondrosarcoma cells, the destruction of the surrounding bone matrix is mediated by catabolic enzymes such as matrix metalloproteinases (MMPs)^1^. Indeed, HIF-2α overexpression caused substantial upregulation of *MMP1*, *MMP2*, and *MMP9* (Supplementary Fig. 5f) and induced an approximately 3-fold increase in total MMP activity (Supplementary Fig. 5g). In contrast, knockdown of HIF-2α substantially reduced the degradation of underlying matrix (Fig. 4h). Among MMP family members, MMP1 has been associated with the metastatic behavior of human chondrosarcomas^33^. We further confirmed that the expression levels of HIF-2α were positively correlated with the levels of MMP1 in chondrosarcoma biopsies (Supplementary Fig. 5h, i and Supplementary Data 2).

To further corroborate our findings with HIF-2α knockdown, we generated HIF-2α knockout SW1353 cells using a CRISPR/Cas9 system. We verified that the genome editing at the *EPAS1* locus was successful resulting in effective HIF-2α knockout (Fig. 4i, j). HIF-2α knockout effectively suppressed all stemness-relevant behaviors and invasive phenotypes (Fig. 4k–o and Supplementary Fig. 5j). Conversely, HIF-2α overexpression promoted these cancer-related behaviors in chondrosarcoma cells (Supplementary Fig. 6).

### *IDH* mutations induce stabilization of HIF-2α in chondrosarcoma cells

**Fig. 5:**
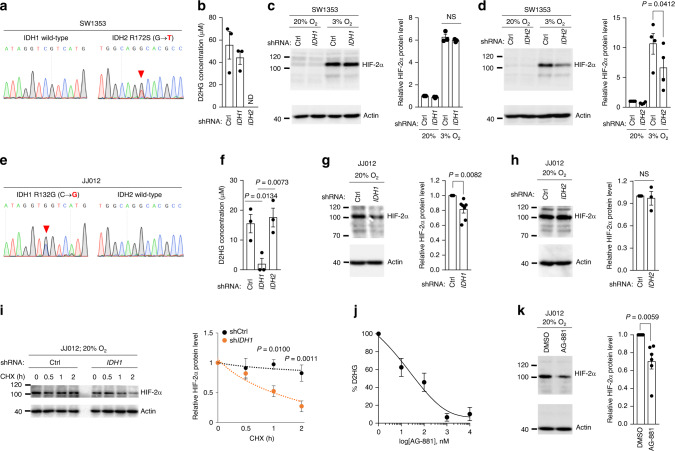
*IDH* mutation causes stabilization of HIF-2α in chondrosarcoma cells. **a** Sanger sequencing confirmed the heterozygous mutation of *IDH2* R172S (AGG > AGT) in SW1353 cells. Red arrowhead indicates the mutated site in the *IDH2* sequence. **b** Intracellular D2HG levels in SW1353 cells harboring the indicated shRNA (*n* = 3). ND; not detected. **c** Immunoblot analysis of HIF-2α protein in SW1353 cells harboring Ctrl or *IDH1* shRNA (*n* = 3). **d** Immunoblot analysis of HIF-2α protein in SW1353 cells harboring Ctrl or *IDH2* shRNA (*n* = 4). **e** Sanger sequencing confirmed the heterozygous mutation of *IDH1* R132G (CGT > GGT) in JJ012 cells. Red arrowhead indicates the mutated site in the *IDH1* sequence. **f** Intracellular D2HG levels in JJ012 cells harboring the indicated shRNA (*n* = 3). **g** Immunoblot analysis of HIF-2α protein in JJ012 cells harboring Ctrl or *IDH1* shRNA (*n* = 7). **h** Immunoblot analysis of HIF-2α protein in JJ012 cells harboring Ctrl or *IDH2* shRNA (*n* = 3). **i** Cycloheximide (CHX) chase analysis of HIF-2α protein in JJ012 cells harboring Ctrl or *IDH1* shRNA. Immunoblots against HIF-2α and actin (left panel) and densitometric analysis of HIF-2α band intensities, normalized against HIF-2α level at the initial time point of each condition (*n* = 6, right panel). **j** Relative D2HG level in JJ012 cells treated with different concentrations of AG-881. The levels were normalized to vehicle treatment (*n* = 3). **k** Immunoblot analysis of HIF-2α protein in JJ012 cells treated with 2 μM AG-881 for 72 h. Representative images for immunoblot analysis (left panel) and relative quantification of HIF-2α protein level (*n* = 6, right panel). Actin was used to verify equal loading of the samples. Data represent mean ± SEM. *P*-values are from one-way ANOVA (**c**, **d**, **f**), two-tailed *t* test (**g**, **h**, **k**), or two-way ANOVA (**i**). NS, not significant. (**c**, **d, g**–**i**, **k**) Full-size immunoblot images are provided in Supplementary Fig. 9.

*IDH* mutations in catalytic arginine residues have been correlated with the induction of pseudohypoxia^17^. Therefore, we investigated whether the *IDH* mutation status in chondrosarcoma cell lines affects HIF-2α expression. First, we verified that SW1353 cells harbor a point mutation at R172 of *IDH2* (Fig. 5a). Knockdown of *IDH2* alleles, but not *IDH1* alleles, in SW1353 abolished the accumulation of D2HG and reduced the expression level of HIF-2α (Fig. 5b–d and Supplementary Fig. 7a). Similarly, we verified a point mutation at R132 of *IDH1* in JJ012 cells, and *IDH1* knockdown, not *IDH2* knockdown, inhibited D2HG accumulation, reducing the level of HIF-2α protein under normoxia (Fig. 5e–h and Supplementary Fig. 7b). We further assessed the stability of HIF-2α by cycloheximide chase analysis of protein degradation. The knockdown of *IDH1* alleles in JJ012 significantly accelerated the degradation of HIF-2α, markedly diminishing the stability of HIF-2α (Fig. 5i). We then utilized a small molecule, AG-881^34,35^, that selectively inhibits the mutant forms of IDHs. Treatment of JJ012 cells with AG-881 effectively abolished D2HG accumulation and significantly decreased HIF-2α expression level (Fig. 5j, k). Meanwhile, *IDH1* and *IDH2* are not mutated in the OUMS-27 cell line^36^; consequently, siRNA-mediated knockdown of *IDH1* or *IDH2* in these cells did not affect HIF-2α levels (Supplementary Fig. 7c). Taken together, *IDH* mutation in chondrosarcoma is implicated conferring increased stability to HIF-2α.

### Combined treatment with HIF-2α inhibitor and chemotherapy agents effectively blocks chondrosarcoma malignancy

Chemoresistance is a major hurdle for clinical management of chondrosarcoma^2^. Several molecular approaches targeting oncogenic signaling pathways mediated by EGFR^37^, mTOR^38,39^, and Src^40,41^ have been successfully attempted to sensitize chondrosarcoma cells to anti-tumor agents, such as cisplatin or doxorubicin. We, therefore, determined whether pharmacological inhibition of HIF-2α activity could represent an option for targeted therapy of chondrosarcoma. The small molecule HIF-2α inhibitor TC-S7009^42^ effectively abolished the transcriptional activity of HIF-2α in chondrosarcoma cells without exerting any discernible cytotoxicity or affecting their migration rate (Supplementary Fig. 8a–c). TC-S7009 treatment effectively blocked sphere formation, clonogenicity, invasive phenotypes, and matrix-degrading activity in SW1353 cells (Supplementary Fig. 8d–g).

**Fig. 6:**
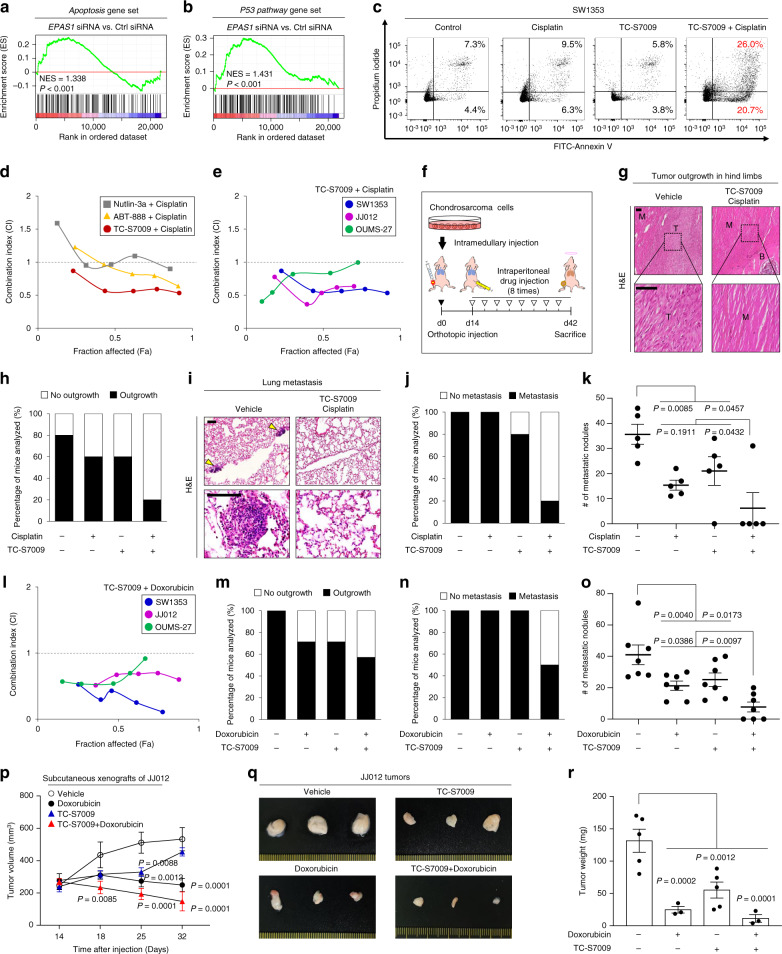
Pharmacological inhibition of HIF-2α enhances the efficiency of chemotherapy for chondrosarcoma. **a**, **b** GSEA was performed with **a** *Apoptosis* and **b** *P53 pathway* gene set using transcriptome data of SW1353 cells transfected with control or *EPAS1* siRNA. NES and nominal *P*-value are indicated. **c** Percentage of apoptotic cells after indicated treatments. Gating strategy is shown in the Supplementary Fig. 10. **d**, **e** Combination index (CI) scores were defined as additive (CI = 1), synergistic (CI < 1), and antagonistic (CI > 1). **d** CI for combinatorial treatment of cisplatin with indicated small molecules in SW1353 cells (*n* = 3). **e** CI for cisplatin and TC-S7009 co-treatments in SW1353 (*n* = 3), JJ012 (*n* = 3), and OUMS-27 (*n* = 8) cells. **f** Schematic illustration of combination chemotherapy with TC-S7009 in orthotopic xenograft of SW1353 cells. **g**–**k** Mice were intraperitoneally injected with vehicle, cisplatin, TC-S7009, or cisplatin plus TC-S7009 (*n* = 5). **g** Representative histology illustrating the extent of local invasion of the transplanted SW1353 cells into the surrounding muscle after indicated treatments. M, muscle; T, tumor; B, bone. Scale bars: 50 μm. **h** Percentage of mice bearing extraosseous outgrowth. **i** Pulmonary metastasis after indicated treatments. Arrowheads indicate metastatic region. Scale bars: 100 μm. **j** The percentage of mice bearing lung metastases and **k** the number of metastatic foci. **l** CI for doxorubicin and TC-S7009 co-treatments in SW1353 (*n* = 8), JJ012 (*n* = 4), and OUMS-27 (*n* = 4). **m**–**o** Mice were injected with indicated drugs (*n* = 7). Percentage of mice bearing **m** extraosseous outgrowth and **n** lung metastases and **o** the number of metastatic foci. **p**–**r** Growth of JJ012 tumor in the subcutaneous xenograft model following injections of indicated drugs (*n* = 5, Vehicle; 3, Doxorubicin; 5, TC-S7009; 3, TC-S7009 + Doxorubicin). **p** Tumor volume at the indicated days after transplantation. **q** Gross images and **r** the weight of excised chondrosarcoma tumors at the end of the treatments. Data represent mean ± SEM. *P*-values are from one-way (**k**, **o**, **r**) or two-way ANOVA (**p**). NS, not significant.

GSEA indicated that HIF-2α knockdown, not HIF-1α knockdown, results in positive enrichment of the *Apoptosis* and *P53 pathway* gene sets (Fig. 6a, b, Supplementary Fig. 8h and Supplementary Tables 10, 11), which are directly related to apoptotic pathways^43^. However, TC-S7009 alone did not noticeably affect the cell death of SW1353 cells (Fig. 6c). Treatment with cisplatin alone resulted in only a minor increase in FITC-Annexin V and propidium iodide positive populations (Fig. 6c), supporting the notion that chondrosarcoma cells are highly resistant to cisplatin^44^. In contrast, combinatorial treatment with TC-S7009 and cisplatin markedly increased the fraction of FITC-Annexin V-positive cells (Fig. 6c), suggesting a synergistic effect of these two agents in triggering apoptosis in SW1353 cells. To further explore the synergism between HIF-2α inhibition and cisplatin, we adopted the Chou–Talalay method to calculate the combination index (CI), which allows quantitative characterization of synergy with drug combinations^45^. ABT-888, a PARP inhibitor, and Nutlin-3a, a p53 activator, were used as positive controls in light of their well-characterized enhancement of cisplatin-induced apoptosis in various cancer cell types^46,47^. The cisplatin–TC-S7009 and cisplatin–ABT-888 combinations exhibited more pronounced synergistic suppression of cell viability than the cisplatin–Nutlin-3a combination in SW1353 cells (Fig. 6d). The synergistic effect of cisplatin–TC-S7009 on cell death were similarly observed in two other chondrosarcoma cell lines, JJ012 and OUMS-27 (Fig. 6e). Additionally, the potential in vivo therapeutic effects of an HIF-2α inhibitor in combination with cisplatin were assessed in orthotopic tumor xenografts. Mice with established SW1353 tumors were treated with vehicle, cisplatin, TC-S7009, or cisplatin in combination with TC-S7009 (Fig. 6f). Cisplatin or TC-S7009 treatment alone caused a mild-to-moderate reduction in extraosseous outgrowth and pulmonary metastatic nodule formation. However, in mice that received the combined therapy of cisplatin and TC-S7009, invasive outgrowth of primary tumors into the muscle tissue surrounding the tibia was nearly abolished and profound suppression of lung metastasis was observed (Fig. 6g–k).

Aside from cisplatin, doxorubicin is one of the most commonly used anti-tumor agents in chondrosarcoma chemotherapy^48,49^. The combinatory effects of HIF-2α inhibition with doxorubicin were evident in the SW1353, JJ012, and OUMS-27 chondrosarcoma cell lines (Fig. 6l), supporting the notion that HIF-2α inhibition sensitizes chondrosarcoma cells to apoptosis induced by DNA-damaging agents. The therapeutic effects of the combined doxorubicin and TC-S7009 treatments was also assessed in the orthotopic mouse model of chondrosarcoma. Mice with established SW1353 tumors were treated with vehicle, doxorubicin, TC-S7009, or doxorubicin in combination with TC-S7009. The combined therapy of doxorubicin and TC-S7009 effectively suppressed extraosseous outgrowth of the primary tumors and significantly reduced pulmonary metastasis (Fig. 6m–o and Supplementary Fig. 8i, j).

Finally, we explored the efficacy of the combined therapy in chondrosarcoma treatment using a subcutaneous tumor model of JJ012 cells. As JJ012-derived chondrosarcoma tumors reached 250 mm^3^ in size, the mice were divided into four groups and treated with vehicle, doxorubicin, TC-S7009, or doxorubicin in combination with TC-S7009 in a therapeutic scheme that did not significantly affect the mouse body weight (Supplementary Fig. 8k). Tumors in the TC-S7009- or doxorubicin-treated groups displayed a delay in tumor growth and lower tumor weight compared to the vehicle control (Fig. 6p–r). Meanwhile, coadministration of doxorubicin and TC-S7009 inhibited the growth of JJ012 tumors even more noticeably, resulting in the smallest tumor size and weight among the tested groups (Fig. 6p–r). Our results collectively demonstrate the efficacy of HIF-2α-targeted therapy in combination with conventional chemotherapy in treating chondrosarcoma.

## Discussion

Chondrosarcomas range from low-grade tumors with virtually no metastatic potential to high-grade tumors characterized by metastatic spread and recurrence. The aggressive forms of chondrosarcomas often become treatment-refractory and lead to a poor prognosis. Several molecular insights into chondrosarcoma pathogenesis have emerged over the past decade^14,18–20^ which may contribute to the timely development of new molecular targeted therapeutics^8^.

Here, we assessed the system-level properties of whole transcriptomes of patients with chondrosarcoma. Gene co-expression analysis often provides a useful global perspective on perturbed transcriptional networks in cancer^50^. This system-wide transcriptome analysis can be further used to construct a roadmap for the identification of key therapeutic targets for diseases^51^. By conducting WGCNA^22^ in patient transcriptomes, we extracted a characteristic gene module potentially responsible for chondrosarcoma malignancy and identified HIF-2α as a master regulator of the module.

We observed that high expression of HIF-2α was associated with high-grade chondrosarcoma tumors. A positive correlation between HIF-2α levels and histological grades of chondrosarcoma in patients was also noted by Liu and collagues^52^. In this study, we further demonstrated that HIF-2α plays a central role in the transition of chondrosarcomas to a highly proliferative, metastatic, and treatment-refractory state. Specifically, HIF-2α endowed chondrosarcoma cells with a high propensity for osteolytic invasion by inducing the expression of matrix-degrading proteases, which are of particular importance in the destruction of bone matrix and enhancing invasiveness^53,54^. These findings are in line with the increased expression of these proteins in metastatic chondrosarcomas^55^. Moreover, our results indicate that HIF-2α promotes tumor growth at primary sites and initiation at metastatic sites by conferring chondrosarcoma cells self-renewal and clonogenic abilities. This is supported by previous studies that report the aberrant stabilization of HIF-2α in association with acquisition of cancer stemness in brain and other neural tumors^56,57^. Furthermore, acquisition of stemness might be implicated in the development of therapy resistance and in facilitating recurrence^31,56^, another critical feature associated with poor survival in patients with chondrosarcoma^1,2^.

The most frequent mutations in chondrosarcoma occur at the catalytic arginine residues of *IDH* genes (*IDH1* and *IDH2*)^14,18^. Therefore, the functions of mutant IDHs are of great interest in understanding the pathogenesis of chondrosarcoma pathogenesis. In this study, we showed that these *IDH* mutations are directly implicated in the stabilization of HIF-2α in chondrosarcoma cells. Recently, Alman and colleagues have linked cholesterol biosynthesis to the tumorigenesis of *IDH* mutation-driven chondrosarcoma^58^. Our transcriptome analysis indicated that genes involved in the cholesterol synthesis pathway were significantly regulated by HIF-2α in chondrosarcoma cells. Together, HIF-2α may serve as a downstream effector that mediates the pathological functions of *IDH* mutations in chondrosarcomas. Aside from IDHs, it has been reported that SIRT1, which specifically promotes HIF-2α stability in several cellular contexts^59^, is frequently overexpressed and promotes metastasis in patients with chondrosarcoma^60^, suggesting another plausible upstream mechanism leading to HIF-2α upregulation. Nonetheless, in light of the significant heterogeneity of chondrosarcoma, comprehensive elucidation of the mechanisms underlying HIF-2α activation is warranted.

Chemoresistance presents a major obstacle for clinical management of chondrosarcoma, limiting the efficacy of conventional anti-cancer therapies^2^. We demonstrated that HIF-2α inhibition in conjunction with conventional chemotherapy agents, cisplatin and doxorubicin, effectively alleviates chondrosarcoma malignancy through their synergistic effects on augmenting chondrosarcoma cell death. Two recently developed TC-S7009 analog HIF-2α antagonists have been shown to have potential for clinical use in clear cell renal cell carcinoma involving elevated HIF-2α levels^61,62^. HIF-2α may, therefore, be a potential druggable target and our findings suggest an intriguing possibility of developing therapeutic strategies for chondrosarcoma patients with HIF-2α hyperactivity.

Nonetheless, several limitations to their potential clinical translation to human patients should be noted. The therapeutic efficacy of HIF-2α inhibitors may be limited by their capacity to penetrate into chondrosarcoma tumors, as they are generally surrounded by thick layers of bone and cartilage matrix. It has been shown that drug delivery to chondrosarcomas is more difficult than to other vascularized cancers, as the drugs are required to diffuse over a longer distance to reach these tumors^63,64^. Therefore, before they can be successfully applied in a clinical setting to patients, there may be several technical barriers that must be overcome to ensure that HIF-2α-targeting small molecules can be efficiently delivered to chondrosarcomas. Another potential limitation of our study includes the use of tumor xenograft models with two different chondrosarcoma cell lines, which may have not sufficiently accounted for the heterogeneity of chondrosarcomas. Further investigations using a large panel of patient-derived tumor xenograft models may be needed to account for the heterogeneity of chondrosarcoma and to reliably predict the disease response to the therapies.

Our study provides proof of concept evidence that inhibiting HIF-2α signaling suppresses progression of chondrosarcoma and improves the efficacy of chemotherapy at cellular and pre-clinical levels. Taken together, we believe that our findings provide molecular insights for the development of anti-cancer therapies to target chondrosarcomas.

## Methods

### Human samples

Human specimens were obtained from the National Cancer Institute Cooperative Human Tissue Network (CHTN). Written informed consents were obtained from all participants by the CHTN. Tissue arrays of various cartilage tumors (T261a) and normal cartilage and chondrosarcoma (OS805) were obtained from the US Biomax, Inc. Histological or immunohistochemistry staining images were acquired with the DS-Ri2 camera (Nikon) connected to an upright microscope. All histological and immunohistochemical specimens were evaluated independently by two pathologists with specific expertise in human sarcomas. The pathologists were blinded to the labeling of the specimens.

### Mice and experimental systems

Female athymic nude mice (BALB/c nu/nu, 4-week old) were purchased from Daehan-Biolink Co. Mice were maintained in the following conditions: temperature: 23–25 °C, relative humidity: 45–65%, and light cycle: 12 h light/12 h dark cycle. Animals were randomly allocated to each experimental group and the treatment allocation was blinded. In the orthotopic injection model, SW1353 or JJ012 cells were trypsinized and resuspended in phosphate-buffered saline (PBS) after centrifugation. The number of cells was adjusted to 1 × 10^6^ cells per 25 μl. For SW1353 cells that stably overexpress eGFP or HIF-2α, the cell number was adjusted to 1 × 10^5^ cells per 25 μl. The cells were mixed with 25 μl of Geltrex (Thermo Fisher Scientific, Geltrex™ LDEV-Free Reduced Growth Factor Basement Membrane Matrix). Prior to the injection procedure, the mice were anesthetized. The skin was incised using a scalpel and a single 0.35-mm diameter hole was drilled through the cartilage of the upper right tibia with the aid of sterile H-files (MANI, 28 mm #70). Subsequently, 50 μl of cell mixture was slowly injected with a 30-gauge microsyringe needle through the hole, which was then sealed using surgical wax and the surgical site was extensively rinsed with sterile PBS to wash out any spilled cell mixture. Lastly, the cutaneous wound was sutured. After the experimental period, the mice were sacrificed, and both hindlimbs were dissected. For lung specimens, PBS was flushed through the trachea and the specimens were processed for histomorphometric and immunohistochemical analysis. For the combined therapy studies using cisplatin and TC-S7009, 2 weeks after the orthotopic injection with chondrosarcoma cells, mice were injected intraperitoneally with or without 2 mg kg^−1^ cisplatin in the presence or absence of 20 mg kg^−1^ TC-S7009 dissolved in Kolliphor EL (castor oil, 10% v/v). For the combined therapy studies using doxorubicin and TC-S7009, 2 weeks after the orthotopic injection with the chondrosarcoma cells, mice were injected intraperitoneally with or without 2 mg kg^−1^ doxorubicin in the presence or absence of 20 mg kg^−1^ TC-S7009 dissolved in Kolliphor EL. The vehicle solution was composed of DMSO (10% v/v), Kolliphor EL (10% v/v), and PBS. The chemotherapy cocktails or the vehicle solution was administered to mice twice weekly for 4 weeks. For the subcutaneous xenograft mouse model, JJ012 cells or JJ012 cells stably transduced with control or *EPAS1* shRNA were trypsinized and resuspended in serum-free DMEM (WelGENE Inc., LM001-05) after centrifugation. The number of cells was adjusted to 5 × 10^6^ cells per 100 μl, which were mixed with 100 μl of Matrigel (Corning, LDEV-free Matrigel basement membrane matrix) and subcutaneously injected into the right flank of nude mice. For the combined therapy, once the tumors were established, mice were randomly divided into four groups for the administration of drugs. Tumor volume was calculated by the following formula: tumor volume = (length × width^2^)/2.

### Cell culture

Chondrosarcoma cell lines SW1353 and OUMS-27 were purchased from ATCC and JCRB Cell Bank, respectively. Chondrosarcoma cell line JJ012 was kindly provided by Professor Joel A. Block (Rush University Medical Centre, Chicago, USA). The cells were maintained in 20 or 3% O_2_ at 37 °C and cultured in DMEM supplemented with 10% FBS (Gibco, 26140079) and 1% antibiotics. For the selection of stable cell lines, 1 × 10^3^ cells were seeded in 100-mm dish and incubated for approximately 2 weeks until the colonies became visible and were supplied with fresh media every 3 days. Visible colonies were individually isolated using cloning rings (Corning). Single colony-forming cells were trypsinized, seeded into 24-well culture plate, and gradually expanded to a 100-mm dish. Transfection was performed with METAFECTENE PRO (Biontex, T040) or jetPRIME® (Polyplus Transfection, 144-07) according to the manufacturer’s protocol. The small interfering RNAs (siRNAs; Bioneer) used are listed in Supplementary Table 12. Cell lines were tested and authenticated using short-tandem repeat profiling by the Korean Cell Line Bank and CosmoGenetech Inc. and were verified to be free of mycoplasma contamination. None of the cell line stocks used in this study were found in the database of commonly misidentified cell lines listed by International Cell Line Authentication Committee.

### Analysis of *IDH1* and -*2* mutation status

Mutation analysis for *IDH*s on two chondrosarcoma cell lines, SW1353 and JJ012, was performed by means of PCR amplification and direct Sanger sequencing of the PCR products. At harvest, cells were washed twice with PBS, and 300 μl lysis buffer was added. The lysis buffer consisted of 100 mM NaCl, 10 mM Tris (pH 8.0), 25 mM EDTA (pH 8.0), 0.5% SDS, and 0.2 mg ml^−1^ proteinase K (Amresco). The cells in the lysis buffer were transferred to the test tube and incubated at 56 °C overnight. The DNA was extracted by adding 300 μl of phenol:chloroform:isoamyl alcohol solution. Next, the DNA was precipitated with 20 μl of 3 M NaAc (pH 5.5) and sequentially washed with 100 and 70% ethanol. The supernatant was removed, and the excessive ethanol was allowed to evaporate for 5 min, after which the DNA pellet was resuspended in 100 μl Tris-EDTA buffer (pH 8.0). Using PCR, 100 ng of genomic DNA (gDNA) fragments of the *IDH1* and *IDH2* loci were amplified. The primer sequences are listed in Supplementary Table 12. The PCR products were purified using the PCR purification kit (MG MED Inc.) according to the manufacturer’s protocol. The purified products were sequenced by Sanger sequencing (Macrogen Inc.) using the same primers as in PCR and the results were analyzed using SnapGene Viewer 4.2.6 (https://www.snapgene.com).

### Histology, immunofluorescence, and immunohistochemistry

Mouse bone tissue samples were fixed in 4% paraformaldehyde in PBS, and decalcified in 0.5 M EDTA (pH 7.4) for 2 weeks, or in 8% nitric acid for 8 h, followed by neutralization in 5% sodium sulfate solution overnight. Lung tissues were fixed in 4% paraformaldehyde solution in PBS at 4 °C overnight. All samples were dehydrated in a graded ethanol series, embedded in paraffin, and sectioned at 5-μm thickness. The number of metastatic foci was counted from ten sections on average, for each mouse. For histological analysis or immunostaining, sections were deparaffinized in xylene and rehydrated. The sectioned samples were stained with hematoxylin and eosin (H&E) or Safranin-O. For immunostaining, antigens were retrieved upon incubation in 10 mM citrate buffer (pH 6.0) or Tris-EDTA buffer (10 mM Tris base, 1 mM EDTA, pH 9.0) at 60 °C for 1 h. Samples were blocked with 1% BSA in PBS and incubated with the primary antibodies at 4 °C overnight. For immunofluorescence, samples were incubated with the secondary antibody at room temperature for 1 h, followed by a 10 min incubation with 1 μg ml^−1^ DAPI. For immunohistochemistry, samples were incubated with 0.3% H_2_O_2_ at room temperature for 5 min. Tissue samples were then incubated with the biotinylated secondary antibodies at room temperature for 1 h, followed by a 30 min incubation with streptavidin-HRP. The signal was developed using an alcohol-soluble chromogen, aminoethyl carbazole (AEC; DAKO). The tissues were stained with Mayer’s hematoxylin (DAKO) for 30 s and placed in 0.08% NH_4_OH for 30 s. The histological or immunohistochemical staining images were acquired with an upright microscope and analyzed. For immunofluorescence detection, the immunostained tissues were imaged with a laser scanning confocal microscope (Carl Zeiss, LSM700). Each chondrosarcoma biopsy was individually examined and graded by the state-certified pathologists at US Biomax according to the World Health Organization guidelines^65^. According to the histological scoring system described in Pirker et al.^66^, the immunohistochemistry score (Hirsch index, H-index) was calculated with the formula: 1 × (percentage of cells staining weakly [1 +]) + 2 × (percentage of cells staining moderately [2 +]) + 3 × (percentage of cells staining strongly [3+]).

### Reagents and antibodies

The HIF-2α inhibitor TC-S7009 (cat. No. 5243) was obtained from Tocris. Kolliphor EL (cat. No. C5135), cycloheximide (cat. No. C7698), cisplatin (cat. No. C2210000), and Nutlin-3a (cat. No. SML0580) were purchased from Sigma Aldrich. The mutant IDH inhibitor AG-881 (cat. No. 28462), ABT-888 (cat. No. 11505) and doxorubicin (cat. No. 15007) were purchased from Cayman. MG132 (A2585) were purchased from Apexbio. Anti-HIF-2α antibody (cat. No. sc-13596), anti-Lamin B antibody (cat. No. sc-6216), anti-MMP1 antibody (cat. No. sc-21731), anti-Actin antibody (cat. No. sc-1615), anti-BrdU antibody (cat. No. sc-32323), normal mouse IgG (cat. No. sc-2025), and goat anti-mouse IgG-B (cat. No. sc-2039) were purchased from Santa Cruz Biotechnology. Anti-Ki67 antibody (cat. No. ab15580) was purchased from Abcam. Anti-human mitochondria antibody (cat. No. MAB1273) was purchased from Millipore. Anti-HIF-2α antibody (cat. No. NB100-122) was purchased from Novus Biologicals. Biotin-SP-conjugated goat anti-rabbit IgG (cat. No. 711-065-152), Dylight 488-conjugated anti-mouse IgG + IgM (cat. No. 315-485-044), peroxidase goat anti-mouse IgG (cat. No. 115-035-044), and peroxidase goat anti-rabbit IgG (cat. No. 111-035-003) were purchased from Jackson ImmunoResearch Labs. Anti-rabbit IgG Alexa Fluor 488 (cat. No. A-21206), anti-rabbit IgG Alexa Fluor 647 (cat. No. A-31573), anti-mouse IgG Alexa Fluor 594 (cat. No. A-21203), and anti-goat IgG Alexa Fluor 488 (cat. No. A-11055) were purchased from Thermo Fisher Scientific.

### Transcriptomics and chromatin immunoprecipitation sequencing analysis

SW1353 cells were transfected with the control, *HIF1A*, or *EPAS1* siRNAs. Three biological replicates were used for each group. cDNA was synthesized using the GeneChip Whole Transcript Amplification kit (Affymetrix) as described by the manufacturer. Microarray service was provided by Macrogen Inc. and performed using the GeneChip® Human Gene 2.0 ST Array (Affymetrix). Array data export processing and analysis were performed using the Affymetrix® GeneChip Command Console® (AGCC) Software. To determine differentially expressed genes between comparison samples, |log_2_(fold change)| > 0.263 and *P*-value (paired *t* test)< 0.05 were used as cut-offs. Publicly available HIF-2α ChIP-Seq datasets (GSM3417828, GSM3417842)^30^ were analyzed using the algorithms STAR^67^ and HOMER^68^ for reads alignment, peak calling, and motif filtering.

### Weighted gene co-expression network analysis

The weighted network construction was performed using the WGCNA R package from Bioconductor, as described by Langfelder and Horvath^22^. The WGCNA builds directed co-expression networks of the genes across microarray samples. The expression profiling analyzed in this study was obtained from 17 freshly frozen chondrosarcoma biopsies (GSE12475)^23^. Nodes of the network correspond to genes and a pair of nodes was connected through an edge if a co-expression relationship existed between them. The network was visualized using Cytoscape software^69^. Network connections of the top 59 M1 module genes with topological overlap above the threshold of 0.13 were visualized using VisANT^70,71^. The hub genes were located toward the network center.

### IPA of patient transcriptome data

IPA (Qiagen, https://www.qiagenbioinformatics.com/products/ingenuity-pathway-analysis/) was used to conduct the disease and function analysis, as well as to identify upstream regulators. The algorithms developed for the use in IPA have been described by Krämer et al.^24^ To analyze the enrichment *P*-value and activation z-score of cancer-relevant gene sets, the diseases and functions analysis of IPA was performed on M1 (263 genes), M2 (265 genes), M3 (418 genes), L1 (1451 genes), L2 (3459 genes), and L3 (3343 genes) module datasets. The regulator-effector analysis was performed to identify upstream regulators of the M1 module. For calculation of the activation z-score, module membership values from WGCNA results were used as inputs.

### Gene set enrichment analysis

Normalized enrichment score (NES) was calculated using the Broad Institute GSEA software, which considers NES = actual ES/mean (ES against all permutations of the dataset). The gene set was considered to be significantly enriched if NES had a false discovery rate *q*-value < 0.25. *P*-value represents the nominal *P*-value of the ES. A nominal *P*-value < 0.05 was considered significant. GSEA was implemented using the GSEA software v.3.0 (https://software.broadinstitute.org/gsea/downloads.jsp). *Cancer stem cell* gene set was obtained from IPA. *Multicancer invasiveness signature* gene set, *Cell migration* gene set, and Hallmark gene sets (*Apoptosis*, *P53 Pathway*) were obtained from MSigDB (https://software.broadinstitute.org/gsea/msigdb/index.jsp).

### Gene Ontology term enrichment analysis

GO term enrichment analysis was performed using Enrichr^72,73^. The top ten most enriched annotations of combined score were selected and represented in bar graphs. The combined score is a combination of the *P*-value and activation z-score calculated by multiplying the two scores as follows:

1 $$c = {\rm{log}}\left( P \right) \times z,$$

where *c* is the combined score, *P* is the *P*-value computed using Fisher’s exact test, and z is the z-score computed to assess the deviation from the expected rank.

### Principal component analysis (PCA)

Based on the canonical HIF-2α target gene expression profiles, PCA was performed using the prcomp R package to predict the HIF-2α activation state in the chondrosarcoma patients. The chondrosarcoma patients were clustered into two groups using the *k*-means clustering based on the values of the principal component axis that represents the HIF-2α activation state.

### Drug interaction studies

For the analysis of the CI, SW1353, JJ012, and OUMS-27 cells were pre-treated with vehicle, 10 μM Nutlin-3a, 10 μM ABT-888, or 10 μM TC-S7009 for 48 h, and additionally treated with cisplatin, doxorubicin, or left untreated for 72 h. The dose of cisplatin ranged between 0.1 and 5 μg ml^−1^, and that of doxorubicin was between 0.01 and 2 μg ml^−1^. Cell viability was measured by the MTT assay. CI was calculated using the Compusyn software version 1.0 and the synergistic effects were determined by the Chou–Talalay method^45^. Drug combination at a non-constant ratio was used to calculate CI. For the analysis of apoptosis, SW1353 cells were pre-treated with 10 μM TC-S7009 or DMSO (vehicle) for 48 h and additionally treated with 5 μg ml^−1^ cisplatin or double distilled water (vehicle) for 24 h. Subsequently, the cells were stained with the FITC-Annexin V Apoptosis Detection kit (Sigma Aldrich, APOAF) according to the manufacturer’s protocol. The cells were then stained with the FITC-Annexin V Apoptosis Detection kit (Sigma Aldrich, APOAF) according to the manufacturer’s protocol. Flow cytometry analysis was performed on a FACS Canto II flow cytometer (BD Biosciences).

### Copy number alteration analysis

Genomic profiles were analyzed using the Gain and Loss Analysis of DNA software (GLAD package from Bioconductor) as described by Hupe et al.^26^. A label (amplification positive or negative) was assigned to each region based on its median DNA copy number. Patients whose smoothing value of a specific gene region was >0 were defined as amplification positive, otherwise they were defined as amplification negative.

### Lentivirus production and transduction

For the construction of the vector overexpressing HIF-2α, the human HIF-2α coding region in the pcDNA3-*EPAS1* plasmid (Addgene, plasmid #18950) was subcloned into the pLJM1-e*GFP* vector (Addgene, plasmid #19319). For the construction of vectors to be used in the RNA interference assays, the sequences of scramble or *EPAS1* short hairpin RNA (shRNA)^74^ were inserted into the pLKO.1 puro plasmid (Addgene, plasmid #8453). The primer sequences used for cloning are listed in Supplementary Table 12. For knockdown of *IDH1 or IDH2* genes in chondrosarcoma cell lines, pGIPZ lentiviral shRNA targeting human *IDH1* (clone No. 320103) or *IDH2* (clone No. 387076) was purchased from GE Dharmacon. The pGIPZ vector, into which the shRNA targeting firefly luciferase was cloned, was kindly gifted from Professor Chanhee Kang (Seoul National University, Seoul, South Korea) and was used as a control vector against pGIPZ-*IDH1* or *IDH2*. To knockout *EPAS1* in SW1353, specific sgRNA sequence targeting the first exon of *EPAS1* (5′-AGGCTGTCAGACCCGAAAAG-3′) was selected using the CRISPOR search algorithm (https://crispor.tefor.net/crispor.py). LentiCRISPR reagents were constructed using lentiCRISPRv2 (Addgene plasmid #52961, as a kind gift from Professor Chanhee Kang from Seoul National University), according to the protocol described by Addgene^75,76^. The lentiCRISPRv2 vector, which does not contain guide RNA, was used as a control. The primer sequences used for cloning are listed in Supplementary Table 12. To produce lentiviruses, HEK293T cells were seeded at 6.5 × 10^6^ cells per 100-mm dish and incubated for 24 h. The vectors for overexpression, knockdown, or knockout were co-transfected with the psPAX2 (Addgene, plasmid #12260) and pMD2.G (Addgene, plasmid #12259) vectors. After 3 days, supernatants were harvested and filtered using 0.45-μm filters. For lentiviral transduction, cells were infected with lentiviruses for 24 h in the presence of 8 μg ml^−1^ polybrene (Sigma Aldrich). Infected cells were selected using 1 μg ml^−1^- puromycin for 3 days.

### T7 endonuclease 1 (T7E1) assay

gDNA was extracted from SW1353 cells transduced with lentiCRISPRv2 lentiviruses. From the extracted gDNA, PCR was performed to amplify the target sequences. The primer sequences used for amplification of the target site are listed in Supplementary Table 12. The PCR amplicons were denatured by heating and annealed slowly to form the heteroduplex DNA. The reannealed heteroduplex DNA was then treated with ten units of mismatch-sensitive T7E1 (New England Biolabs) at 37 °C for 20 min and analyzed using 2% agarose gel electrophoresis. The mutation frequencies were calculated based on the band intensities using ImageJ software (National Institutes of Health) and the following equation: mutation frequency (%) = 100 × (1 − (1−fraction cleaved)^1/2^), where the fraction cleaved is the total relative density of the cleavage bands divided by the sum of the relative density of the cleavage bands and uncut bands^77^.

### Cycloheximide chase analysis

JJ012 cells harboring control or *EPAS1* shRNA were treated with 100 μM of cycloheximide for the indicated number of hours prior to lysis. The protein samples were subjected to immunoblot for analysis of protein stability. The band intensity was measured using ImageJ and normalized to the band intensity of Actin.

### Transwell invasion assay

The invasion assay was performed using the Transwells (BD Falcon, pore size, 8 μm, PET) in a 24-well plate. Filters were coated with 20 μg of Geltrex in 20 μl of serum-free DMEM and dried for 4 h in a sterile environment. Prior to performing the invasion assay, SW1353, JJ012, and OUMS-27 cells were starved for 12 h in serum-free DMEM. Immediately before seeding cells, the dried transwells were rehydrated with 25 μl of serum-free DMEM at 37 °C for 1 h. Starved cells were trypsinized, centrifuged, and resuspended in serum-free DMEM. In each transwell, 1 × 10^5^ cells were placed in 200 μl of serum-free DMEM and 2 ml of DMEM supplemented with 10% FBS was added to the 24-well plate. The plate was incubated at 37 °C for 48 h, fixed in 4% paraformaldehyde for 2 min, permeabilized in 100% methanol for 20 min, and stained with 0.1% crystal violet in 10% ethanol for 20 min. Cells on the upper side of the transwell were removed with cotton-tipped swabs. During each of the staining steps, the transwells were washed with PBS. The underside of the transwells was examined under a microscope and images were taken using the DS-Ri2 camera. Image fields were randomly chosen, and the number of invading cells was counted using the ImageJ software.

### Sphere formation assay

SW1353, JJ012, and OUMS-27 cells were suspended in DMEM-F12 medium or DMEM-F12 medium supplemented with 2% of B-27 supplement (Thermo Fisher Scientific), 20 ng ml^−1^ epithermal growth factor (EGF; Invitrogen), and 20 ng ml^−1^ basic fibroblast growth factor (bFGF; Peprotech). For experiments using SW1353 cells overexpressing eGFP or HIF-2α, cells were suspended in DMEM-F12 medium in the absence of the above supplements. SW1353 and OUMS-27 cells were then seeded at a density of 1 × 10^4^ cells per well, and JJ012 cells were seeded at a density of 2 × 10^3^ cells per well in a 24-well Ultra-low attachment plate (Corning). After 4 days, fresh medium was added to each well. Seven days later, the spheres were analyzed by sphere diameter and the number of spheres larger than 50 μm was counted. Three representative fields per well were imaged (Axio Observer Z1, Zeiss) and the diameters were measured using Image-Pro Premier software; the longest three diameters per image field for each well were recorded. The distribution of the sphere diameters was plotted using the notched Box-and-Whisker plot originally invented by Tukey.

### Gelatin degradation assay

SW1353, JJ012, and OUMS-27 cells were seeded onto coverslips coated with Oregon Green 488-conjugated gelatin (Life Technologies) at 4 × 10^4^ cells per well in a 24-well plate. Briefly, coverslips were treated with 50 μg ml^−1^ poly-d-Lysine (Corning) for 20 min followed by 0.5% glutaraldehyde (Junsei Chemical) on ice for 15 min. The treated coverslips were coated with pre-warmed 200 μg ml^−1^ Oregon Green 488-conjugated gelatin at 37 °C for 10 min in the dark, treated with 5 mg ml^−1^ NaBH_4_ for 15 min, and extensively washed with 70% ethanol and PBS. The cells were incubated for 18 h after seeding. For imaging the gelatin degradation, cells were fixed with a 50% methanol (v/v) and 10% acetic acid (v/v) solution for 10 min and stained with 1 μg ml^−1^ DAPI. For each coverslip, at least three fields were imaged at ×20 magnification using a fluorescence microscope. To quantify gelatin degradation, the area of degradation in each field was measured by the color-thresholding, using the ImageJ software.

### Luciferase assay

SW1353 cells were transfected with 1 μg of a firefly luciferase vector containing hypoxia responsive element repeats. The thymidine kinase promoter-Renilla luciferase reporter plasmid (pRL-TK) was used as a control for the transfection efficiency. For HIF-2α overexpression, the pcDNA3-*EPAS1* (Addgene) plasmid was transfected using METAFECTENE PRO. Twenty-four hours after transfection, the cells were treated with TC-S7009 for an additional 24 h. Luciferase activity was measured using the luciferin substrate and normalized by the Renilla luciferase activity.

### Migration assay

SW1353, JJ012, and OUMS-27 cells were cultured at a density of 5 × 10^5^ per well in a 6-well plate. When cells achieved 90% confluence, a wound was made using a sterile 200 μl pipette tip. Cells were washed with PBS three times and incubated in fresh media supplemented with 1% FBS for 36 h. Every 12 h, randomly selected fields around the wound area were captured by a digital camera under a microscope (EVOS XL Core Cell Imaging System, Thermo Fisher Scientific).

### Colony formation assay

SW1353 cells were suspended in DMEM supplemented with 10% FBS and seeded in a 6-well plate; 1 × 10^3^ and 500 cells were seeded in each well for the knockdown and overexpression experiments, respectively. The medium was changed every 3 days for 14 days. OUMS-27 cells were suspended in DMEM supplemented with 10% FBS and seeded in a 6-well plate; 5 × 10^3^ and 1 × 10^3^ cells were seeded for the knockdown and overexpression experiments, respectively. The medium was changed every 3 days for 21 days. JJ012 cells were suspended in DMEM supplemented with 10% FBS and seeded in a 6-well plate; 1 × 10^3^ cells were seeded for the knockdown experiment. The medium was changed every 3 days for 10 days. Colonies were fixed in a 4% paraformaldehyde solution for 2 min and permeabilized in 100% methanol for 20 min. Colonies were then stained with 0.1% (w/v) crystal violet solution for 20 min.

### BrdU incorporation assay

For the analysis of cell proliferation, SW1353, JJ012, and OUMS-27 cells were seeded onto coverslips in a 24-well plate at a density of 1 × 10^4^ cells per well. After culturing for 48 h, cells were starved in serum-free DMEM for 24 h. Fresh DMEM supplemented with 10% FBS was then added and cells were incubated for 12 h. For BrdU incorporation, cells were pulsed with 10 μM BrdU for 3 h (SW1353 and JJ012) or 12 h (OUMS-27), and BrdU incorporation was analyzed using fluorescence microscopy (EVOS FL Cell Imaging System, Thermo Fisher Scientific).

### MMP activity assay

Measurement of MMP enzymatic activity was performed using the MMP Activity Assay kit (Abcam) according to the manufacturer’s protocol. Briefly, 1 ml of conditioned media containing MMPs, secreted from SW1353 cells, was collected, and concentrated to 100 μl. The substrate solution (50 μl), which contained the fluorescence resonance energy transfer peptide whose cleavage by MMPs generates green fluorescence, was mixed with a 50 μl aliquot of the sample. Fluorescence intensity was detected kinetically every 5 min for 1 h with excitation at 488 nm and emission at 530 nm, using a Spectramax Gemini microplate fluorescence reader (Molecular Device).

### Measurement of D-2-hydroxygultarate

SW1353 and JJ012 cells transduced with control, *IDH1*, or *IDH2* shRNAs were seeded in 6-well plates at a density of 5 × 10^4^ cells per well and harvested after 48 h. JJ012 cells cultured using the same conditions were treated with AG-881 for 72 h. Cells were then collected and intracellular D2HG was measured in the cell lysates. The intracellular D2HG concentration was measured using a D2HG colorimetric assay kit (Biovision) according to the manufacturer’s protocol.

### Quantitative real-time PCR

Total RNA was isolated using the TRI reagent® (Molecular Research Center, Inc.). The RNA was reverse transcribed using EasyScript Reverse Transcriptase (Transgen Biotech) and qRT-PCR was performed using SYBR Green PCR Master Mix (Enzynomics) with a StepOnePlus Real-Time PCR System (Applied Biosystems). The PCR conditions began with an initial denaturation step of 10 min at 95 °C, followed by 40 cycles of PCR consisting of 20 s at 95 °C and 1 min at 60 °C. The melting curves were collected at the final stage by increasing the temperature from 60 °C to 95 °C. The PCR data was analyzed using the 2^-∆∆CT^ method and *HPRT* was used as the housekeeping gene. The primer sequences are listed in Supplementary Data 4.

### Statistics and reproducibility

The statistical significance of differences between two groups was assessed by a two-tailed unpaired Student’s *t* test. For multiple group comparisons and repeated measures, statistical significance was determined by one-way ANOVA, when appropriate with uncorrected Fisher’s least significant difference or two-way repeated measure ANOVA with Šidák’s post hoc test. Experiments were independently replicated at least three times. Data are expressed as mean ± SEM. *P* < 0.05 was considered statistically significant. Survival curves were estimated based on the Kaplan–Meier method and compared using the log-rank test in 64 chondrosarcoma patients, excluding three people whose information was lost during follow-ups. In transplantation experiments, animals were randomly allocated to each experimental group and the treatment allocation was blinded. For the measurement of correlation between two groups, R and *P*-values were calculated by Spearman’s rank correlation coefficient. To analyze the contingency table, *P-*values were obtained using the chi-square test. Statistical analysis was performed using the SPSS 22 statistical software (IBM) and dot plots and survival curves were plotted using GraphPad Prism 8 software. For the comparison of sphere diameters, Tukey distribution bars were used to emphasize data range distribution and the notches of each box were analyzed. The box and whisker plot in Fig. 4c shows median values (center line) and the 25th (bottom line) and 75th percentiles (top line) with whiskers indicating the range. Outliers are represented by dots. Notch shows 95% confidence interval of the median. Box plots were plotted using the XLSTAT software (Addinsoft). All data were collected from at least three independent experiments.

### Study approval

Before obtaining biopsies, the study was approved by the Institutional Review Board (IRB) of the Seoul National University (IRB No. E1611/003-008 and E1803/003-007). All animal studies were approved by the Seoul National University Institutional Animal Care and Use Committees (IACUC; IACUC Nos. SNU-151202-7-3 and SNU-151216-2-6). Animal experiments were reported in accordance with the ARRIVE guidelines.

### Reporting summary

Further information on research design is available in the Nature Research Reporting Summary linked to this article.

## Supplementary information

Supplementary Information

Description of Additional Supplementary Files

Supplementary Data 1

Supplementary Data 2

Supplementary Data 3

Supplementary Data 4

Reporting Summary

## Data Availability

The expression profiling and genome variation profiling data referenced during the study are available in a public repository from the NCBI (https://www.ncbi.nlm.nih.gov) website. For the transcriptome analysis, the following datasets were used: chondrosarcoma patient dataset (GSE12475), murine chondrocyte dataset (GSE73659), and the primary chondrosarcoma cells dataset (GSE47823). For the copy number alteration analysis, the chondrosarcoma patient dataset (GSE12532) was used. The HIF-2α ChIP-Seq datasets (GSM3417828, GSM3417842) were used for the ChIP-Seq analysis. The original transcriptome datasets that were produced in this study are deposited in the Gene Expression Omnibus (GSE156565). *Cancer stem cell* gene set was obtained from IPA. *Multicancer invasiveness signature*, *Cell migration*, *Apoptosis*, and *P53 Pathway* gene sets were obtained from MSigDB v.6.0. All other relevant data supporting the findings of this study are available within the article and its Supplementary information files or from the corresponding author upon reasonable request.
