# Supplementary material for: A system-level approach identifies HIF-2α as a critical regulator of chondrosarcoma progression
Source: Nat Commun. 2020 Oct 6;11:5023. doi: 10.1038/s41467-020-18817-7 (PMC7538956; doi:10.1038/s41467-020-18817-7)
Supplement: Supplementary file 1 — Supplementary Information [file 41467_2020_18817_MOESM1_ESM.docx]

SUPPLEMENTARY INFORMATION

**A system-level approach identifies HIF-2α as a critical regulator of chondrosarcoma progression**

Kim et al.

**Supplementary Figures 1-10**

**Supplementary Tables 1-12**

**
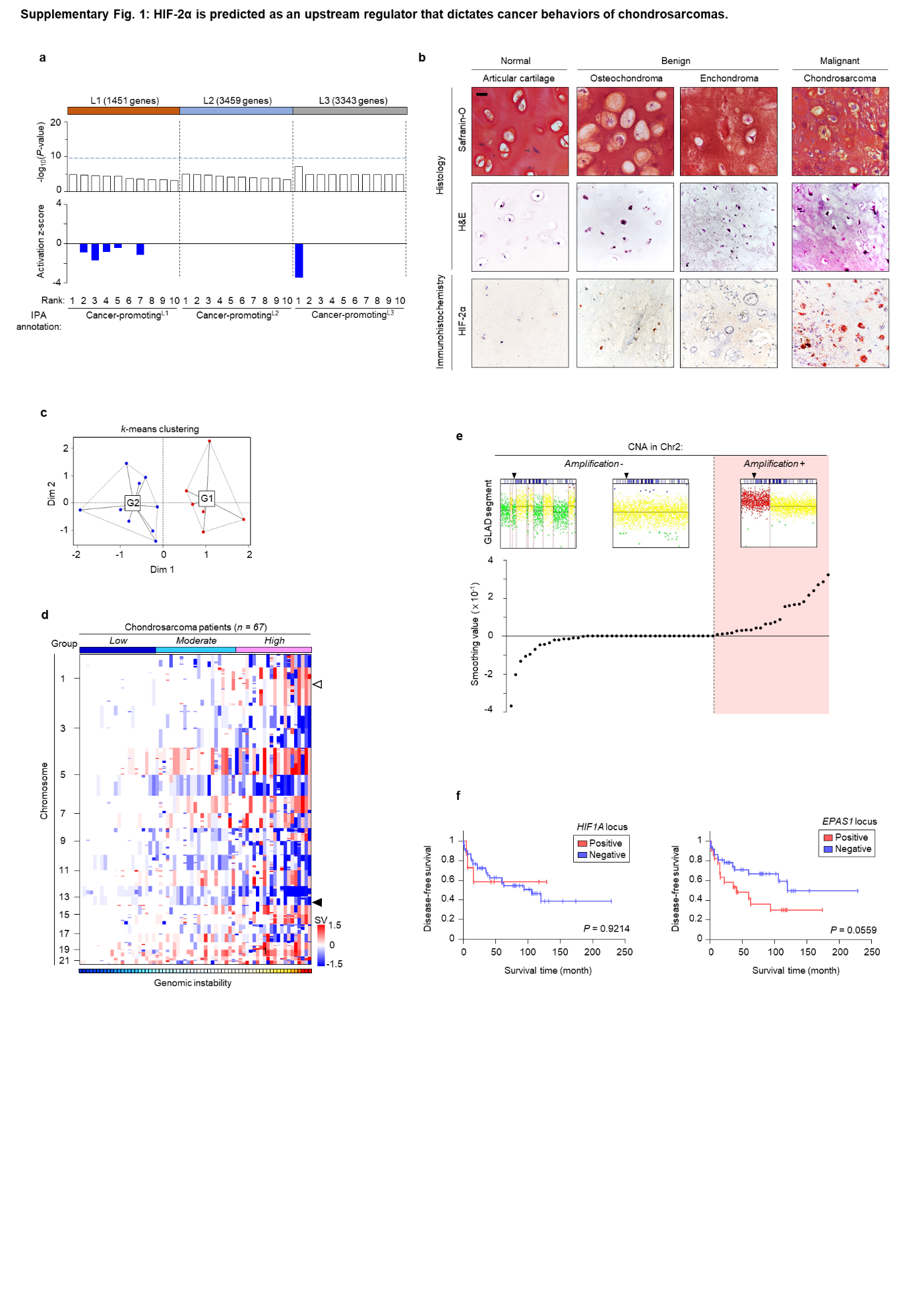
Supplementary figures and figure legends**

**Supplementary Fig. 1: HIF-2α is predicted as an upstream regulator that dictates cancer behaviors of chondrosarcomas.**

**a** Three large gene modules, L1 (brown), L2 (turquoise), and L3 (gray) were identified by WGCNA using transcriptome data of patients with chondrosarcoma (GSE12475). Cancer-promoting (CP) annotations significantly associated with each module were analyzed using IPA. For each module, top 10 CP annotations were arranged in the order of –log_10_(*P*-value). A right-tailed Fisher’s exact test was used to determine the statistical significance. **b** Representative images of Safranin-O staining, H&E staining, and IHC against HIF-2α in normal human articular cartilage, osteochondroma, enchondroma, and chondrosarcoma biopsies. Scale bar: 25 μm. **c** Using chondrosarcoma transcriptome data (GSE12475), chondrosarcoma patients were grouped into two groups (Gs), G1 and G2, by conducting *k*-means clustering based on values of the principal component axis which represents the HIF-2α activation state. Principal component analysis (PCA) was performed based on the collective expression profiles of canonical HIF-2α target genes in chondrosarcoma tissues derived from the patients. The list of canonical HIF-2α target genes retrieved from IPA is listed in Supplementary Table 4. Dim, dimension. **d** Smoothed copy number data for 67 patients with chondrosarcoma are shown. Patients were ordered by interchromosomal variation, a genomic instability indicator. Open and solid arrowheads indicate *EPAS1* and *HIF1A* loci, respectively. **e** Amplification status of 67 chondrosarcoma patients were determined based on smoothing value (SV) at *EPAS1* loci. Inlets are representative genomic profiles for chromosome 2 of patients with amplification negative (–) or positive (+) in the *EPAS1* locus. Solid arrowheads indicate the *EPAS1* position in chromosome 2. Vertical dashed red lines in inlet images represent the breakpoints detected with GLAD; the assigned status is indicated by a color code. Chr2, chromosome 2. **f** Kaplan–Meier plot of disease-free survival of patients stratified by amplification status of *HIF1A* (left panel; positive: *n* = 9; negative: *n* = 55) and *EPAS1* (right panel; positive: *n* = 24; negative: *n* = 40) loci. Significance was calculated with the two-sided log-rank test.

**
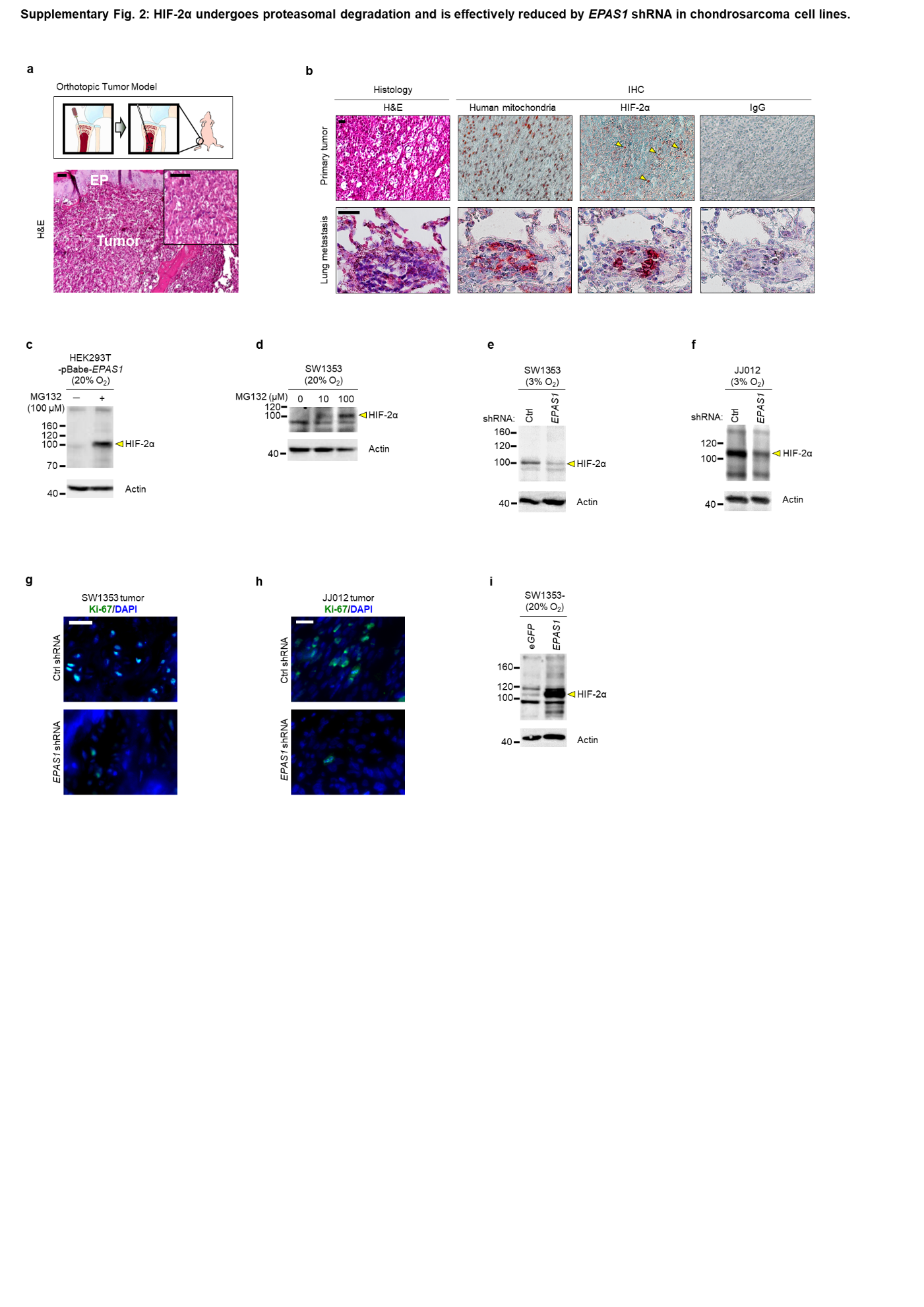
**

**Supplementary Fig. 2: HIF-2α undergoes proteasomal degradation and is effectively reduced by *EPAS1* shRNA in chondrosarcoma cell lines.**

**a** Schematic illustration of the chondrosarcoma orthotopic mouse model (upper panel). Primary chondrosarcoma tumors formed within the tibial intramedullary canal following SW1353 xenograft (1×10^6^ cells). EP; epiphyseal plate (lower panel). Scale bar: 50 μm. **b** Histology and IHC analysis of primary and pulmonary metastatic chondrosarcoma tumors. Arrowheads indicate HIF-2α positive cells. Scale bars: 25 μm. **c**, **d** HIF-2α protein is regulated via proteasome-dependent degradation. MG132 was treated for 4h before lysis. (**c**) Immunoblot of HIF-2α protein in HEK293T cells transfected with pBabe-*EPAS1* in the absence or presence of MG132. (**d**) Immunoblot of endogenous HIF-2α in SW1353 cells treated with indicated dose of MG132. The arrowhead indicates the position of HIF-2α protein. Actin was used to verify equal loading of the samples. **e**, **f** The effect of knockdown by *EPAS1* shRNA. Immunoblot of HIF-2α protein in (**e**) SW1353 and (**f**) JJ012 cells that are transduced with Ctrl or *EPAS1* shRNA. The arrowhead indicates the position of HIF-2α protein. Actin was used to verify equal loading of the samples. **g**, **h** Representative images of IF against Ki-67 in primary tumors established with (**g**) SW1353 or (**h**) JJ012 cells harboring indicated shRNAs. Scale bar: 25 μm. **i** Immunoblot of HIF-2α protein in SW1353 cells overexpressing e*GFP* or *EPAS1*. Arrowhead indicates the position of HIF-2α protein. Actin was used to verify equal loading of the samples. **c**–**f**, **i** Full-size immunoblot images are provided in Supplementary Fig. 9.


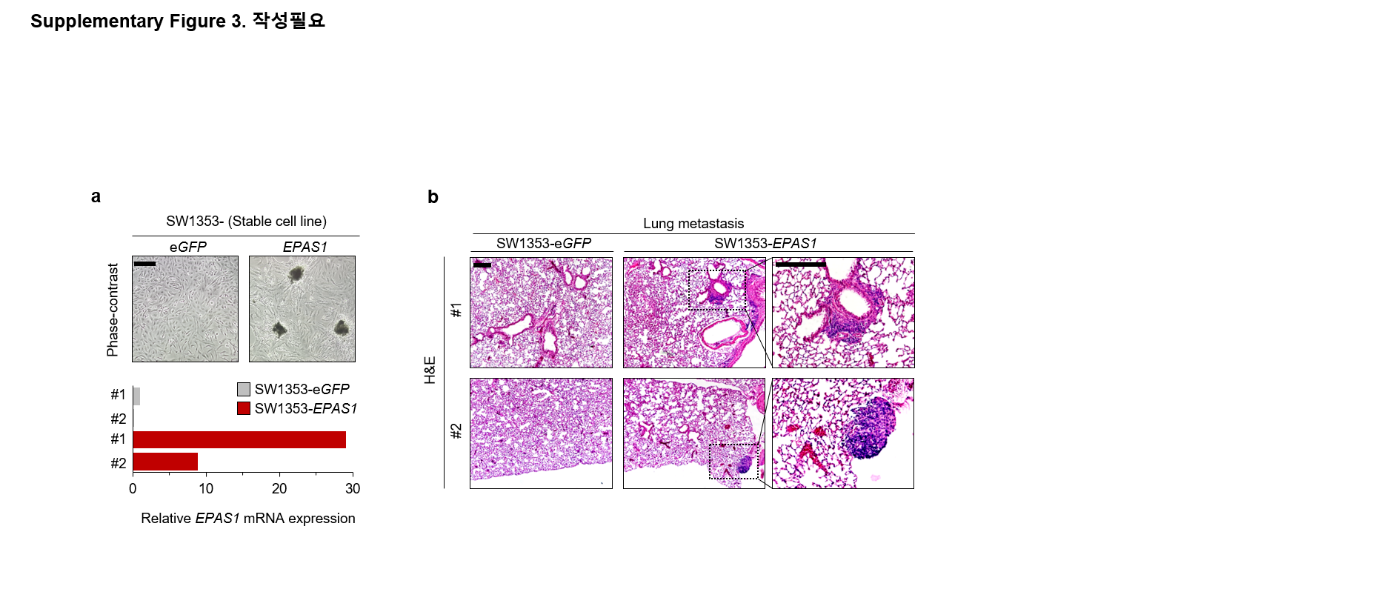


**Supplementary Fig. 3: HIF-2α overexpression in SW1353 tumors promotes cancer progression.**

**a** Representative phase-contrast images of SW1353 stable cell lines overexpressing eGFP or HIF-2α (upper panel). Relative mRNA level of *EPAS1* in the stable cell lines (lower panel). Scale bar: 150 μm. **b** Representative H&E staining images of metastatic nodules of SW1353 cells overexpressing e*GFP* or *EPAS1*. Scale bar: 200 μm.


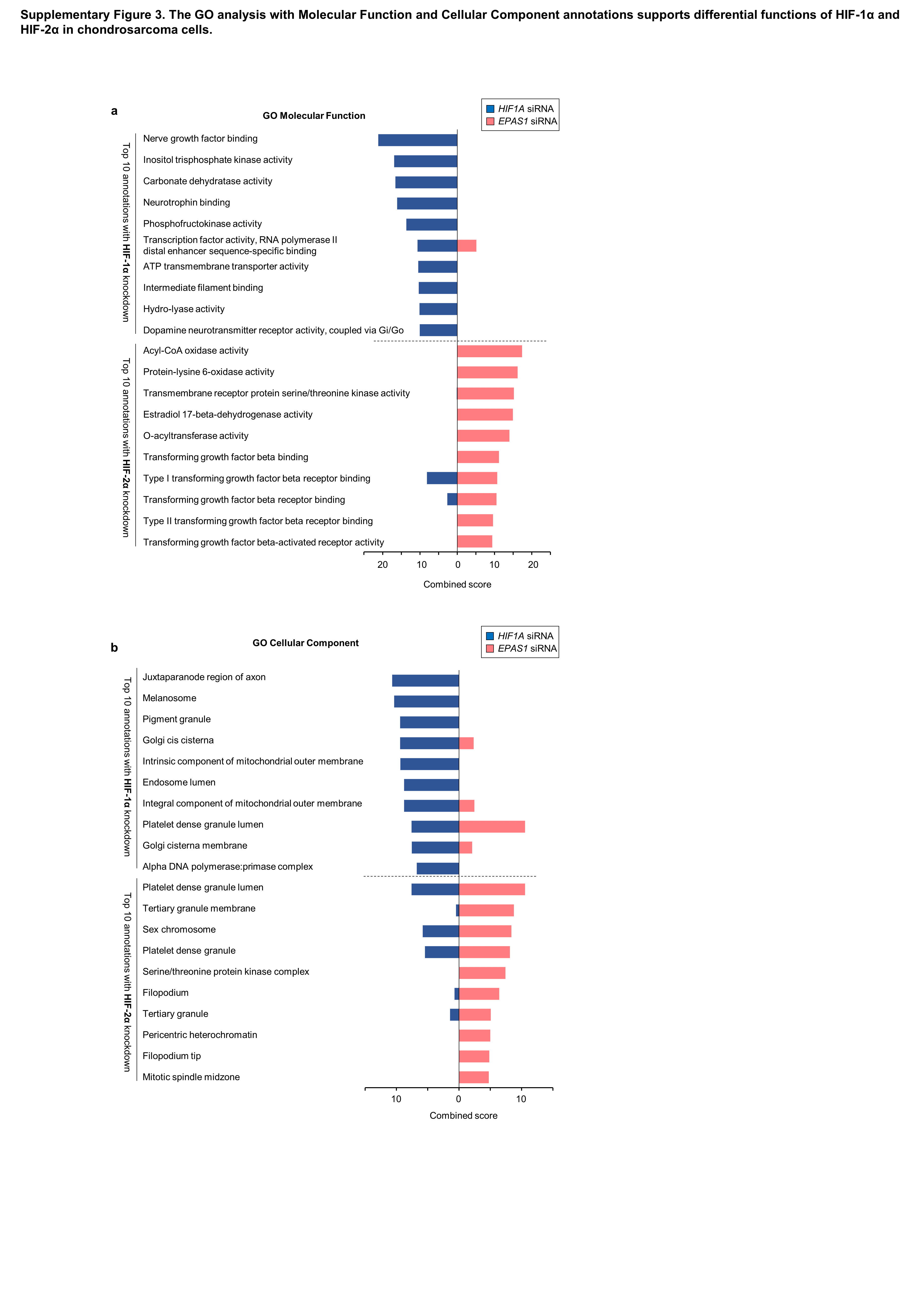


**Supplementary Fig. 4: GO analysis with Molecular Function and Cellular Component annotations supports differential functions of HIF-1α and HIF-2α in chondrosarcoma cells.**

**a**, **b** Top 10-ranked (**a**) Molecular Function and (**b**) Cellular Component GO terms associated with differentially downregulated genes in HIF-1α or HIF-2α knockdown conditions. The combined score reflects a combination of *P*-value computed using two-sided Fisher's exact test and z-score as explicitly described in Methods section.

**
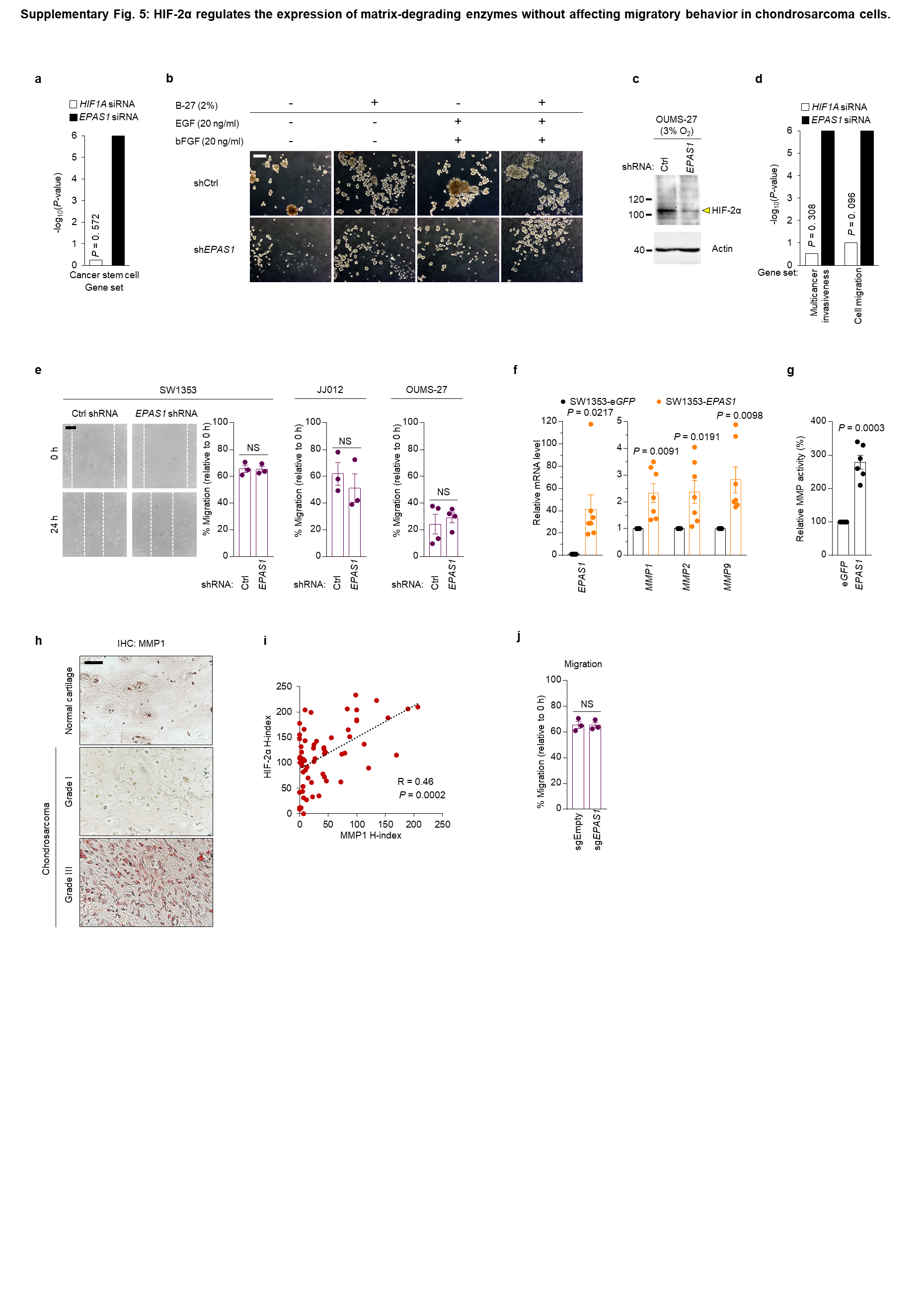
**

**Supplementary Fig. 5: HIF-2α regulates expression of matrix-degrading enzymes without affecting migratory behavior in chondrosarcoma cells.**

**a** Bar graph displays *P*-values from GSEA conducted with *Cancer stem cell* gene set in transcriptome data of SW1353 cells transfected with indicated siRNAs. A –log_10_(*P*-value) of 6 indicates an actual *P*-value of less than 1×10^-6^ (i.e. *P* < 0.000001). **b** Sphere formation assay performed with SW1353 cells transduced with indicated shRNAs. Cells were treated with or without B-27 (2%), EGF (20 ng ml^-1), or bFGF (20 ng ml^-1). Images are representative of three biologically independent experiments. Scale bar: 200 μm. **c** The effect of knockdown by *EPAS1* shRNA. Immunoblot of HIF-2α protein in OUMS-27 cells that are transduced with Ctrl or *EPAS1* shRNA. The arrowhead indicates the position of HIF-2α protein. Actin was used to verify equal loading of the samples. **d** Bar graph displays *P*-values from GSEA conducted with *Multicancer invasiveness* and *Cell migration* gene sets in transcriptome data of SW1353 cells transfected with indicated siRNAs. A –log_10_(*P*-value) of 6 indicates an actual *P*-value of less than 1×10^-6^ (i.e. *P* < 0.000001). **e** Migration assay using SW1353 (representative images, *n* = 3), JJ012 (*n* = 3), and OUMS-27 cells (*n* = 4) expressing indicated shRNAs. Scale bar: 200 μm. **f** Relative mRNA level of indicated genes in SW1353 overexpressing e*GFP* or *EPAS1* (*n* = 7). **g** Relative total MMP activity in SW1353 overexpressing e*GFP* or *EPAS1* (*n* = 6). **h** Representative images of IHC against MMP1 in human normal cartilage and chondrosarcoma biopsies. Scale bar: 100 μm. **i** Correlation between H-index of HIF-2α and that of MMP1 in chondrosarcoma biopsies (*n* = 65). R and *P*-value were calculated based on Spearman’s rank correlation coefficient. **j** Quantitation of migration assay (*n* = 3) in SW1353 cells transduced with lentivirus harboring Cas9 and indicated sgRNAs. Data represent the mean ± SEM. *P*-values are from two-tailed *t*-test (**e**–**g**, **j**). NS, not significant.

**
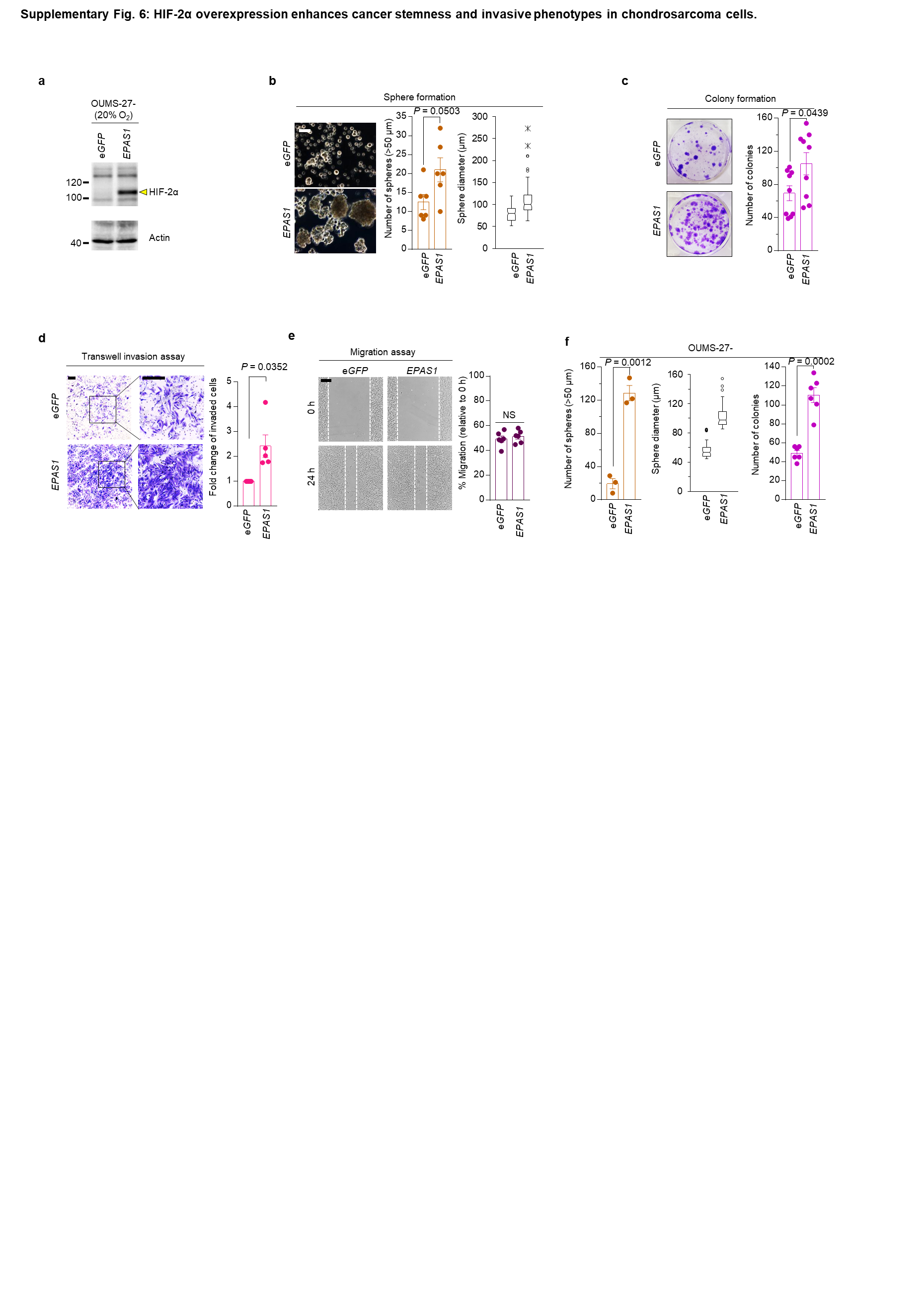
**

**Supplementary Fig. 6: HIF-2α overexpression enhances cancer stemness and invasive phenotypes in chondrosarcoma cells.**

**a** Immunoblot of HIF-2α protein in OUMS-27 cells overexpressing e*GFP* or *EPAS1*. Arrowhead indicates the position of HIF-2α protein. Actin was used to verify equal loading of the samples. **b** Sphere formation assay of SW1353 cells overexpressing e*GFP* or *EPAS1*. Representative images are shown (left panel), and the number (middle panel) and the diameter (right panel) of spheres were measured (*n* = 6). Box and whisker plot shows median values (center line) and the 25^th^ (bottom line) and 75^th^ percentiles (top line) with whiskers indicating the range. Outliers are represented by dots. Notch shows 95% confidence interval of the median. Scale bar: 200 μm. Cells were grown in serum-free condition. **c** Representative images (left panel) and quantitation of colony formation assay by SW1353 cells overexpressing e*GFP* or *EPAS1* (*n* = 9, right panel). **d** Representative images of crystal violet staining from the transwell invasion assay using SW1353 cells transduced with lentiviruses harboring e*GFP* or *EPAS1* (left panel). Quantitation of the number of invaded SW1353 cells in indicated conditions (*n* = 5, right panel). Scale bar: 200 μm. **e** Migration assay was performed using SW1353 overexpressing e*GFP* or *EPAS1*. Images were taken 0 and 24 h after wound formation (left panel) and the migrated area was measured (*n* = 6, right panel). Scale bar: 100 μm. **f** Quantitation of sphere formation assay (*n* = 3, left and middle panel) and colony formation assay (*n* = 6, right panel) using OUMS-27 cells overexpressing e*GFP* or *EPAS1*. Box and whisker plot drawn as in **b**. Data represent mean ± SEM. *P*-values are from two-tailed *t*-test (**b**–**f**). NS, not significant. **a** Full-size immunoblot image is provided in Supplementary Fig. 9.


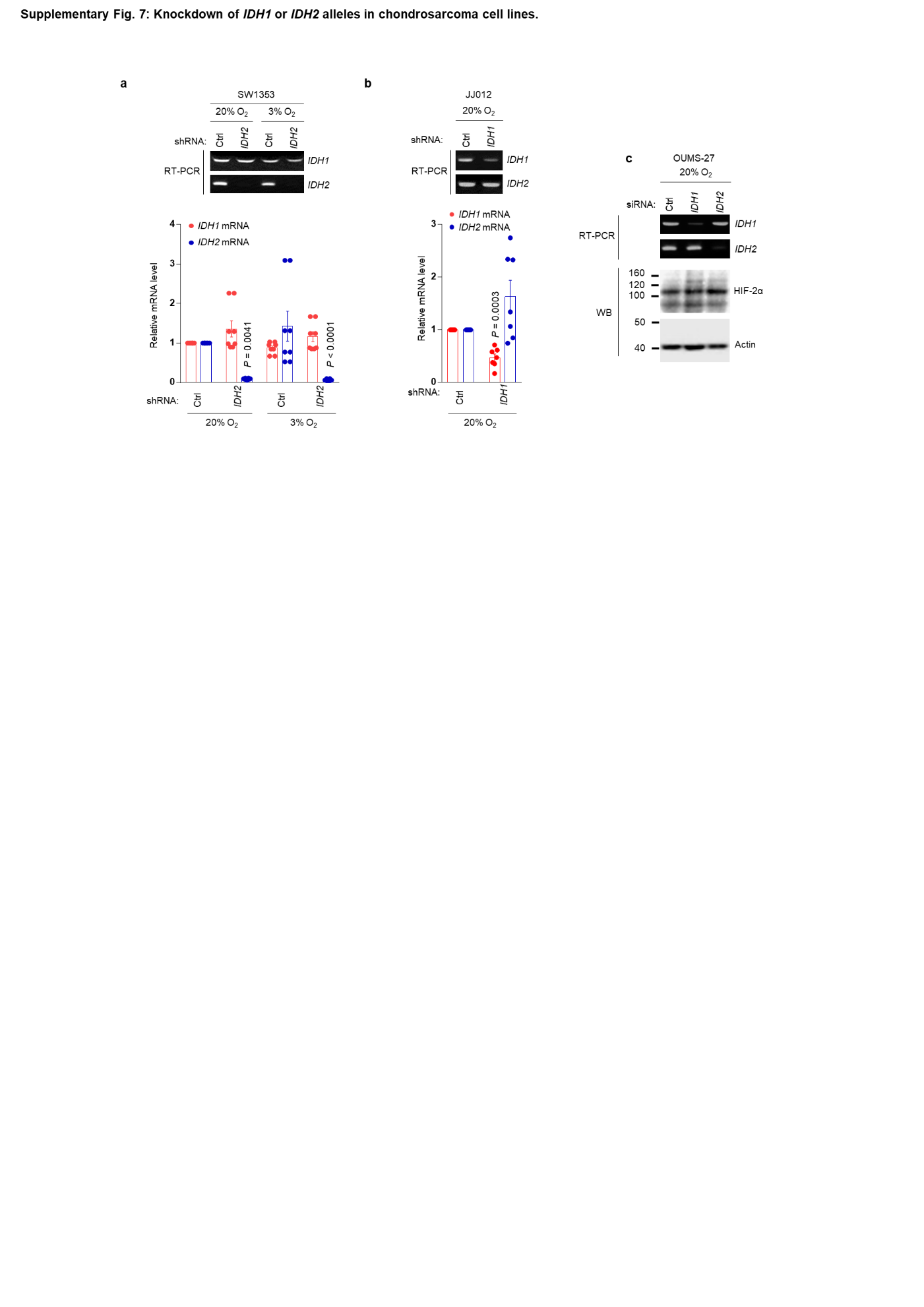


**Supplementary Fig. 7: Knockdown of *IDH1* or *IDH2* alleles in chondrosarcoma cell lines.**

**a** SW1353 cells were stably transduced with Ctrl or *IDH2* shRNA. Cells were passaged and incubated under normoxic (20% O_2_) or hypoxic (3% O_2_) conditions. RT-PCR analysis (upper panel) and qRT-PCR analysis (*n* = 8, lower panel) for *IDH1* and *IDH2* mRNA levels. **b** JJ012 cells were stably transduced with Ctrl or *IDH1* shRNA. Cells were passaged and incubated under normoxia. RT-PCR analysis (upper panel) and qRT-PCR analysis (*n* = 7, lower panel) for *IDH1* and *IDH2* mRNA levels. **c** OUMS-27 cells were transfected with Ctrl, *IDH1*, or *IDH2* siRNA and grown under normoxic (20% O_2_) condition. RT-PCR and immunoblot analysis of *IDH1* and *IDH2* mRNA levels and HIF-2α protein level. Arrowhead indicates position of HIF-2α protein. Actin was used to verify equal loading of the samples. WB; western blot. Data represent mean ± SEM. *P*-values are from two-way ANOVA (**a**), or two-tailed *t-*test (**b**). **a**–**c** Full-size agarose gel and immunoblot images are provided in Supplementary Fig. 9.

**
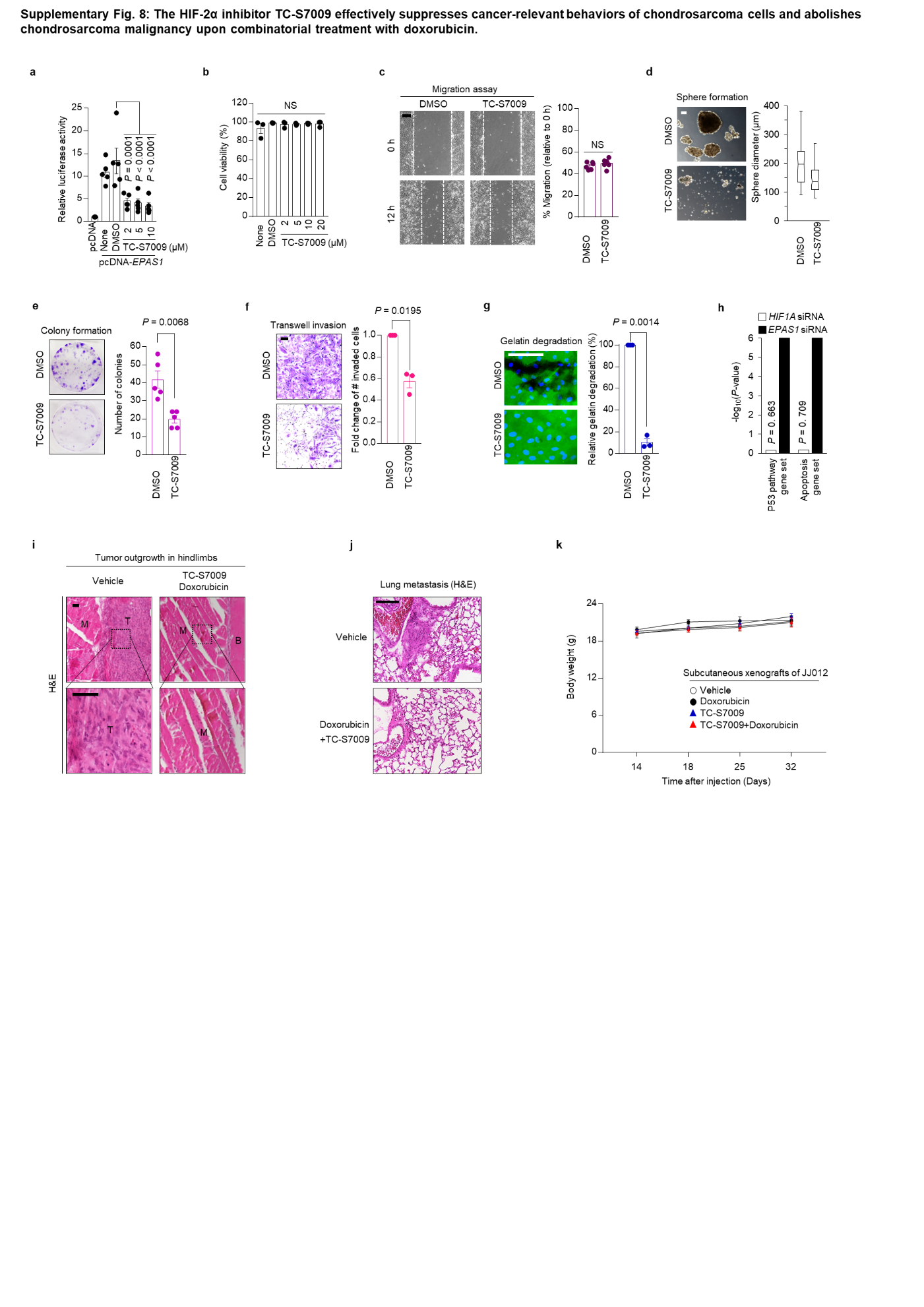
**

**Supplementary Fig. 8. HIF-2α inhibitor TC-S7009 effectively suppresses cancer-relevant behaviors of chondrosarcoma cells and abolishes chondrosarcoma malignancy upon the combinatorial treatment with doxorubicin.**

**a** SW1353 cells co-transfected with pcDNA3 or pcDNA3-*EPAS1* and the hypoxia response element (HRE) containing reporter in the absence or presence of TC-S7009. HRE reporter activity normalized to Renilla luciferase activity (*n* = 5). **b** Trypan blue exclusion assay to test the effect of TC-S7009 on cell viability of SW1353 cells (*n* = 3). **c**–**g** Cell-based assays conducted to assess the effect of HIF-2α inhibitor on tumor-initiating potentials and invasiveness in chondrosarcoma cells. (**c**) migration assay (*n* = 6), (**d**) Sphere formation assay (*n* = 3), (**e**) colony formation assay (*n* = 5), (**f**) Transwell invasion (*n* = 3), and (**g**) gelatin degradation assay (*n* = 3) using SW1353 cells treated with DMSO or 10 μM TC-S7009.Box and whisker plot shows median values (center line) and the 25^th^ (bottom line), and 75^th^ percentiles (top line) with whiskers indicating the range. Outliers are represented by dots. Notch shows 95% confidence interval of the median. Scale bars: 100 μm. **h** Bar graph displays *P*-values from GSEA conducted with *P53 pathway* and *Apoptosis* gene sets in transcriptome data of SW1353 cells transfected with indicated siRNAs. A –log_10_(*P*-value) of 6 indicates an actual *P*-value < 1×10^-6^ (i.e. *P* < 0.000001). **i**, **j** Mice were intraperitoneally injected with vehicle or doxorubicin and TC-S7009 twice a week for four weeks following orthotopic xenograft of SW1353 cells. (**i**) Representative H&E images illustrating the extent of local invasion of the orthotopically transplanted JJ012 cells into muscle tissue surrounding the tibia after the indicated treatments. M; muscle, T; tumor, B; bone. Scale bars: 50 μm. (**j**) Representative H&E image showing the extent of metastatic growth of chondrosarcoma cells in lung after the indicated treatments. Scale bars: 100 μm. **k** JJ012 cells were subcutaneously transplanted into athymic mice. Indicated chemicals were intraperitoneally injected into the xenografted mice two times a week for four weeks. Body weight was measured at the indicated days after tumor transplantation (*n* = 5). Data represent mean ± SEM. *P*-values are from one-way ANOVA (**a**, **b**), two-tailed *t*-test (**c**, **e**–**g**), or two-way ANOVA (**k**). NS, not significant.


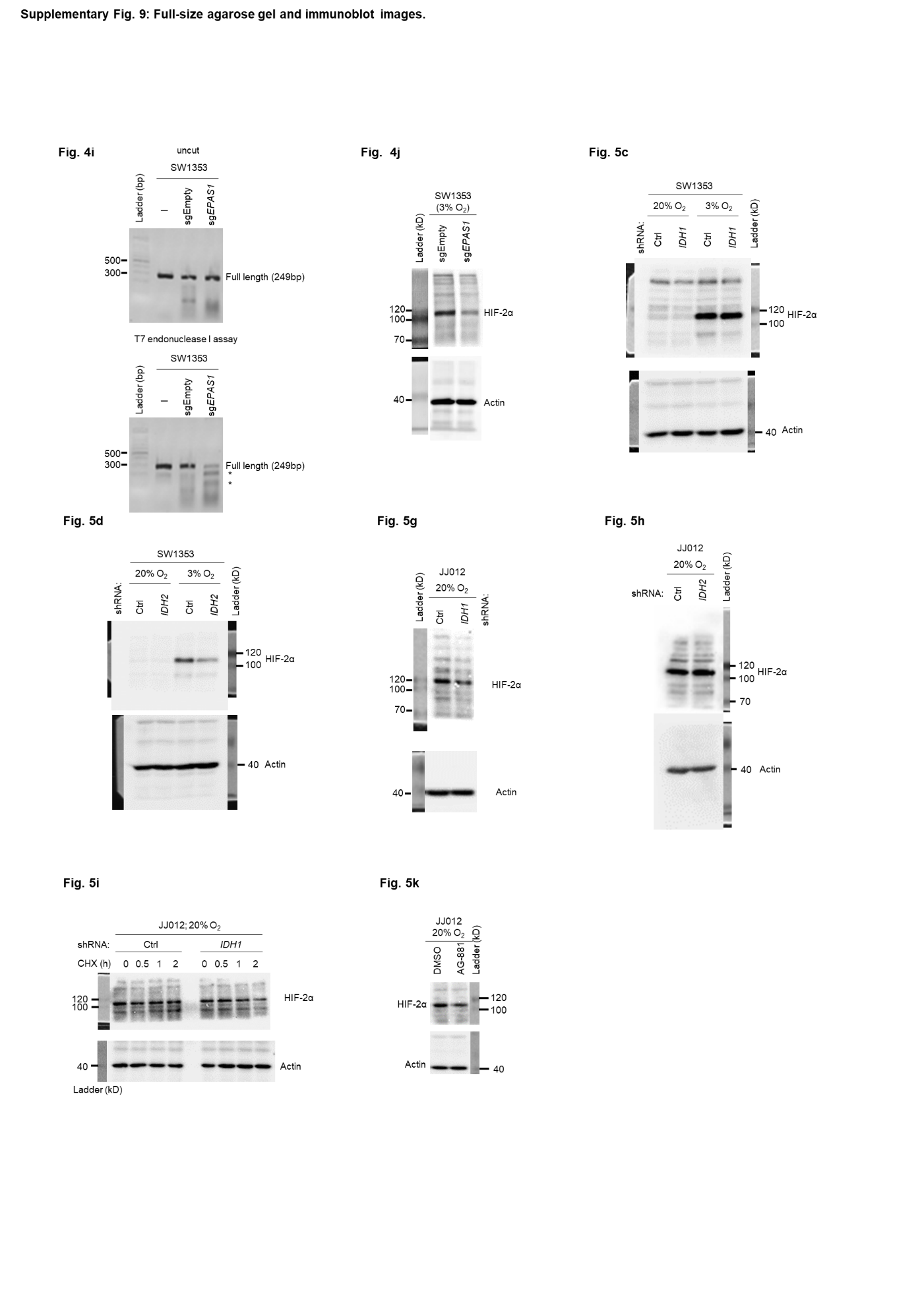


**Supplementary Fig. 9: Full-size agarose gel and immunoblot images.**

**
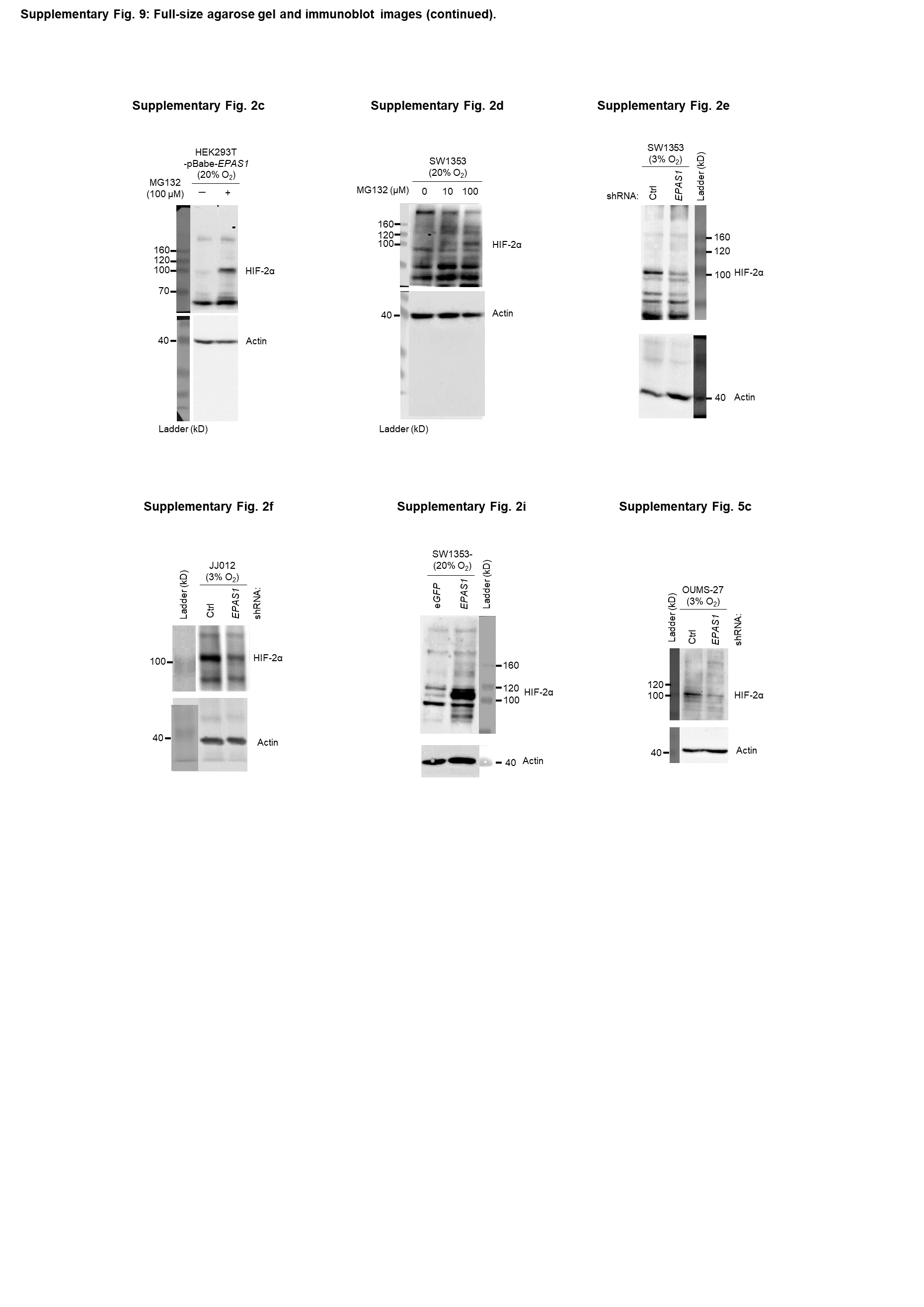
**

**Supplementary Fig. 9: Full-size agarose gel and immunoblot images (continued).**

**
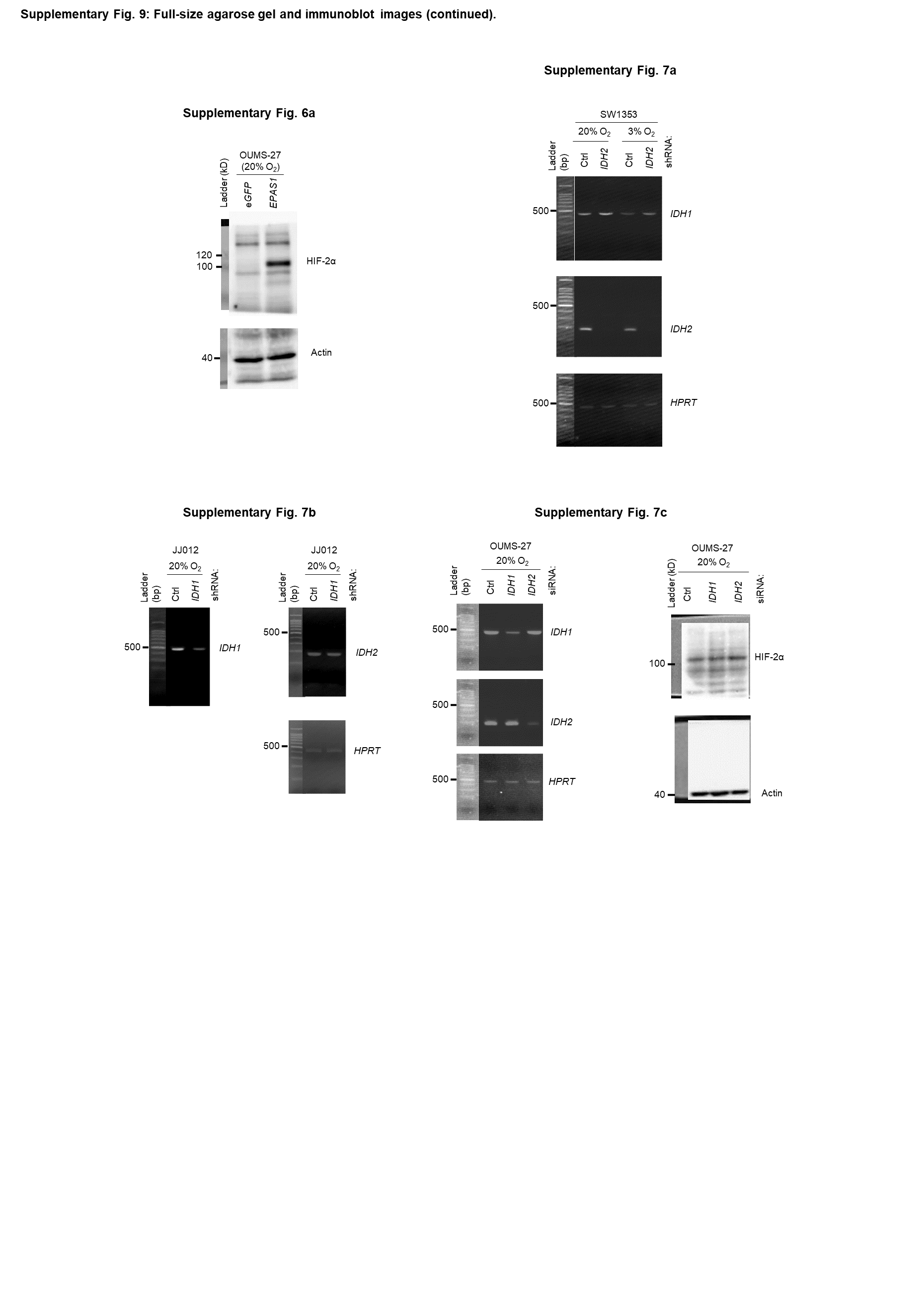
**

**Supplementary Fig. 9: Full-size agarose gel and immunoblot images (continued).**


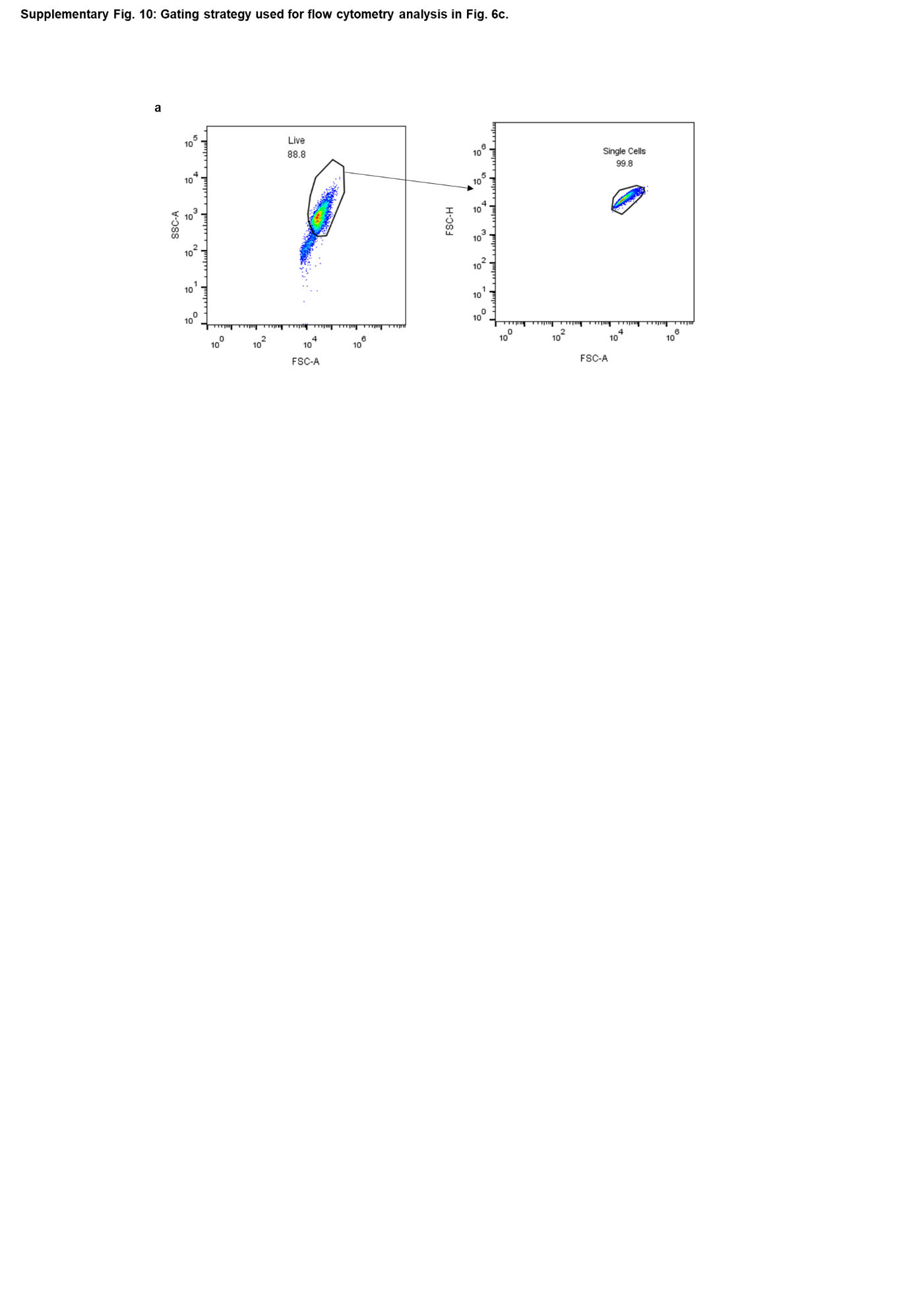


**Supplementary Fig. 10: Gating strategy for flow cytometry analysis in Figure 6c.**

**a** Cells were gated by forward scatter (FSC) and side scatter (SSC) area (A) according to cell size and granularity to remove debris and large clumps. Singlet cells were then selected using FSC-A and FSC-height (H).

**Supplementary Table 1: Analysis of diseases or functions annotation by Ingenuity Pathway Analysis (IPA), referring to Fig. 1a and Supplementary Fig. 1a.**

Top 30 diseases or functions annotations of the M1 (cyan) module.

|  | **Diseases or functions annotation** | ***P*-value** | **Activation z-score** |
| --- | --- | --- | --- |
| 1 | Growth of tumor | 3.87E-19 | 2.536 |
| 2 | Metastasis | 5.08E-16 | 3.356 |
| 3 | Advanced malignant tumor | 1.68E-15 | 3.356 |
| 4 | Breast or colorectal cancer | 2.91E-14 | -0.478 |
| 5 | Malignant solid tumor | 4.19E-12 | 2.35 |
| 6 | Cancer | 4.45E-12 | 2.79 |
| 7 | Central nervous system cancer | 4.79E-11 |  |
| 8 | Abdominal neoplasm | 1.17E-10 | -0.684 |
| 9 | Abdominal cancer | 2.22E-10 | -0.695 |
| 10 | Metastatic solid tumor | 6.22E-10 | 3.246 |
| 11 | Colorectal cancer | 6.33E-10 | -1.067 |
| 12 | Lymphocytic cancer | 8.79E-10 | 0.243 |
| 13 | Head and neck cancer | 1.04E-09 |  |
| 14 | Head and neck neoplasia | 1.5E-09 |  |
| 15 | Advanced malignant solid tumor | 1.76E-09 | 3.246 |
| 16 | Digestive organ tumor | 3.02E-09 | -1.414 |
| 17 | Malignant neoplasm of large intestine | 3.37E-09 | -1.067 |
| 18 | Gastrointestinal tract cancer | 3.45E-09 | -1.067 |
| 19 | Digestive system cancer | 4.77E-09 | -1.129 |
| 20 | Angiogenesis of tumor | 4.77E-09 | -1.258 |
| 21 | Lung tumor | 5.45E-09 | 2.24 |
| 22 | Neoplasia of tumor cell lines | 7.75E-09 | 1.363 |
| 23 | Solid tumor of head and neck | 7.9E-09 |  |
| 24 | Non-melanoma solid tumor | 9.8E-09 | 0.923 |
| 25 | Lung cancer | 2.86E-08 | 1.943 |
| 26 | Lymphocytic neoplasm | 4.61E-08 | 0.57 |
| 27 | Uterine serous papillary cancer | 4.95E-08 |  |
| 28 | Tumorigenesis of tissue | 5.52E-08 | -0.197 |
| 29 | T-cell non-Hodgkin disease | 5.57E-08 |  |
| 30 | Primary central nervous system tumor | 0.000000062 |  |

Top 30 diseases or functions annotations of the M2 (purple) module.

|  | **Diseases or functions annotation** | ***P*-value** | **Activation z-score** |
| --- | --- | --- | --- |
| 1 | Breast or ovarian cancer | 8.79E-08 |  |
| 2 | Mammary tumor | 1.6E-06 |  |
| 3 | Growth of tumor | 1.95E-06 | 1.467 |
| 4 | Invasion of tumor | 2.32E-06 | 2.054 |
| 5 | Advanced malignant tumor | 2.35E-06 | 0.508 |
| 6 | Oral tumor | 3.67E-06 |  |
| 7 | Plasma cell dyscrasia | 3.73E-06 |  |
| 8 | Benign neoplasia | 3.96E-06 | -0.152 |
| 9 | Oral cancer | 4.11E-06 |  |
| 10 | Breast cancer | 4.79E-06 |  |
| 11 | Urogenital cancer | 6.93E-06 |  |
| 12 | Multiple myeloma | 8.16E-06 |  |
| 13 | Oral cavity carcinoma | 1.01E-05 |  |
| 14 | Invasion of tumor cells | 1.03E-05 | 1.798 |
| 15 | Neoplasia of cells | 1.18E-05 | 0.901 |
| 16 | Metastasis | 1.34E-05 | 0.508 |
| 17 | Oral squamous cell carcinoma | 1.91E-05 |  |
| 18 | Cancer | 1.94E-05 | 1.211 |
| 19 | Lymphohematopoietic neoplasia | 3.43E-05 | -0.518 |
| 20 | Genital tract cancer | 3.44E-05 |  |
| 21 | Cancer of cells | 4.14E-05 | 0.475 |
| 22 | Precancerous condition | 4.55E-05 |  |
| 23 | Metastatic solid tumor | 7.89E-05 | 0.13 |
| 24 | Tumorigenesis of genital organ | 8.08E-05 |  |
| 25 | Genital tumor | 8.24E-05 |  |
| 26 | Neoplasia of lymphoid organ | 8.36E-05 |  |
| 27 | Gonadal tumor | 9.53E-05 |  |
| 28 | Hematological neoplasia | 0.000096 | -0.647 |
| 29 | Barrett syndrome | 0.0000992 |  |
| 30 | Upper aero-digestive squamous cell carcinoma | 0.0000994 |  |

Top 30 diseases or functions annotations of the M3 (green) module.

|  | **Diseases or functions annotation** | ***P*-value** | **Activation z-score** |
| --- | --- | --- | --- |
| 1 | Cell death of osteosarcoma cells | 5.99E-05 | -2.828 |
| 2 | Waldenström macroglobulinemia | 0.000795 |  |
| 3 | Early-onset breast cancer | 0.00277 |  |
| 4 | Proliferation of colon carcinoma cells | 0.0044 |  |
| 5 | Cell death of cancer cells | 0.00443 | -2.333 |
| 6 | Cell death of tumor cells | 0.00516 | -2.538 |
| 7 | NRAS Q61 mutation positive metastatic melanoma | 0.0102 |  |
| 8 | NRAS Q61X mutation positive cutaneous melanoma | 0.0102 |  |
| 9 | TNM stage II non-small cell lung cancer | 0.0102 |  |
| 10 | Aggregability of colorectal cancer cell lines | 0.0102 |  |
| 11 | Grade 1 non-small cell lung cancer | 0.0102 |  |
| 12 | Grade 3 non-small cell lung cancer | 0.0102 |  |
| 13 | Grade 4 non-small cell lung cancer | 0.0102 |  |
| 14 | High grade colon tumor | 0.0102 |  |
| 15 | High grade pancreatic tumor | 0.0102 |  |
| 16 | High grade thyroid gland tumor | 0.0102 |  |
| 17 | Recurrent hepatocellular carcinoma | 0.0102 |  |
| 18 | Stage 1A mycosis fungoides | 0.0102 |  |
| 19 | Stage 1B mycosis fungoides | 0.0102 |  |
| 20 | Stage 3c NRAS Q61 mutation melanoma | 0.0102 |  |
| 21 | Hereditary gingival fibromatosis | 0.0113 |  |
| 22 | Stage 3 cancer | 0.0124 |  |
| 23 | Platinum-sensitive recurrent ovarian cancer | 0.0127 |  |
| 24 | Plasma cell dyscrasia | 0.0129 |  |
| 25 | T-cell non-Hodgkin disease | 0.0159 |  |
| 26 | Relapsed B-cell non-Hodgkin lymphoma | 0.0176 |  |
| 27 | Angiogenesis of head and neck tumor | 0.0203 |  |
| 28 | Apoptosis of T cell lymphoma cells | 0.0203 |  |
| 29 | Chemotherapy refractory platinum-sensitive recurrent ovarian cancer | 0.0203 |  |
| 30 | High grade stomach tumor | 0.0203 |  |

Top 10 diseases or functions annotations of the L1 (brown) Module

|  | **Diseases or functions annotation** | ***P*-value** | **Activation z-score** |
| --- | --- | --- | --- |
| 1 | Epithelial cancer | 5.99E-05 |  |
| 2 | Non-melanoma solid tumor | 0.000795 | -0.859 |
| 3 | Malignant solid tumor | 0.00277 | -1.698 |
| 4 | Cancer | 0.0044 | -0.843 |
| 5 | Tumorigenesis of tissue | 0.00443 | -0.44 |
| 6 | Abdominal cancer | 0.00516 |  |
| 7 | Abdominal neoplasm | 0.0102 | -1.129 |
| 8 | Production of tumor | 0.0102 |  |
| 9 | Adenocarcinoma | 0.0102 |  |
| 10 | Digestive system cancer | 0.0102 |  |

Top 10 diseases or functions annotations of the L2 (turquoise) Module

|  | **Diseases or functions annotation** | ***P*-value** | **Activation z-score** |
| --- | --- | --- | --- |
| 1 | Intestinal carcinoma | 1.04E-05 |  |
| 2 | Large intestine adenocarcinoma | 1.41E-05 |  |
| 3 | Gastrointestinal carcinoma | 2.47E-05 |  |
| 4 | Intestinal tumor | 4.58E-05 |  |
| 5 | Intestinal cancer | 8.28E-05 |  |
| 6 | Large intestine neoplasm | 8.51E-05 |  |
| 7 | Gastrointestinal tract cancer and tumors | 0.000116 |  |
| 8 | Gastrointestinal tract cancer | 0.000138 |  |
| 9 | Malignant neoplasm of large intestine | 0.000153 |  |
| 10 | Cancer | 0.000478 | 0.024 |

Top 10 diseases or functions annotations of L3 (gray) Module

|  | **Diseases or functions annotation** | ***P*-value** | **Activation z-score** |
| --- | --- | --- | --- |
| 1 | Cell death of osteosarcoma cells | 7.8E-08 | -3.464 |
| 2 | Grade 2-4 testicular cancer | 1.79E-05 |  |
| 3 | Limited disease stage small cell lung cancer | 1.79E-05 |  |
| 4 | Metastatic poorly differentiated neuroendocrine carcinoma | 1.79E-05 |  |
| 5 | Poorly differentiated small cell lung carcinoma | 1.79E-05 |  |
| 6 | Regional Merkel-cell carcinoma | 1.79E-05 |  |
| 7 | Resectable large-cell carcinoma | 1.79E-05 |  |
| 8 | Resectable small cell lung carcinoma | 1.79E-05 |  |
| 9 | Stage 1 relapsed testicular nonseminomatous germ cell tumor | 1.79E-05 |  |
| 10 | Stage 1 relapsed testicular seminoma | 1.79E-05 |  |

**Supplementary Table 2:** **Results of the upstream regulator analysis of M1-associated cancer-promoting (CP) annotation referring to Fig. 1b.**

| **Upstream regulator** | **CP#** | **Diseases or functions annotation** | | ***P*-value** | | **Activation z-score** | | **The sum of activation z-score** | |
| --- | --- | --- | --- | --- | --- | --- | --- | --- | --- |
| *EPAS1* | CP1 | Invasion of cells | | 1.33E-07 | | 2.426 | | 18.485 | |
| *EPAS1* | CP2 | Invasion of tumor cell lines | | 3.14E-06 | | 2.23 | | 18.485 | |
| *EPAS1* | CP3 | Migration of cells | | 1.93E-05 | | 1.23 | | 18.485 | |
| *EPAS1* | CP4 | Metastasis | | 1.61E-07 | | 1.498 | | 18.485 | |
| *EPAS1* | CP5 | Proliferation of cells | | 4.86E-05 | | 2.08 | | 18.485 | |
| *EPAS1* | CP7 | Lung cancer | | 3.64E-05 | | 1.96 | | 18.485 | |
| *EPAS1* | CP8 | Vasculogenesis | | 4.15E-10 | | 1.592 | | 18.485 | |
| *EPAS1* | CP9 | Cell proliferation of tumor cell lines | | 1.07E-05 | | 1.578 | | 18.485 | |
| *EPAS1* | CP11 | Migration of endothelial cells | | 1.96E-08 | | 1.48 | | 18.485 | |
| *EPAS1* | CP13 | Quantity of connective tissue | | 6.58E-05 | | 1.411 | | 18.485 | |
| *EPAS1* | CP17 | Colony formation of cells | | 2.75E-05 | | 1 | | 18.485 | |
| *HIF1A* | CP1 | Invasion of cells | | 0.000102 | | 1.312 | | 2.734 | |
| *HIF1A* | CP12 | Angiogenesis | | 3.32E-08 | | 1.422 | | 2.734 | |
| *SMARCA4* | CP3 | Migration of cells | | 3.07E-06 | | 2.198 | | 4.18 | |
| *SMARCA4* | CP6 | Cell spreading | | 5.11E-07 | | 1.982 | | 4.18 | |
| *TP63* | CP4 | Metastasis | | 2.58E-06 | | 2.169 | | 4.462 | |
| *TP63* | CP11 | Migration of endothelial cells | | 8.98E-07 | | 1 | | 4.462 | |
| *TP63* | CP15 | Growth of tumor | | 6.75E-07 | | 1.293 | | 4.462 | |
|  | | |  | |  | |  | |  |

**Supplementary Table 3:** **Top 59 genes of M1 with topological overlap above the threshold of 0.13 were analyzed with the VisANT software, referring to Fig. 1d.**

| **First metanode group genes** | | | | | |
| --- | --- | --- | --- | --- | --- |
| Non-target | | | | HIF-2α target | |
| *ADA* | *GADD45G* | *NOTCH4* | *SPHK1* | *CAPG* | *S100A4* |
| *ADGRG1* | *GGA2* | *NPEPL1* | *SPRY1* | *CLIC3* | *SEMA4B* |
| *AES* | *GPR68* | *PARVB* | *ST5* | *DDAH2* | *SERPING1* |
| *C10ORF11* | *GRASP* | *PDGFRB* | *TBX2* | *ENG* | *TEAD2* |
| *CALHM2* | *GSTM4* | *PLTP* | *TCF7L1* | *IER2* | *TMEM173* |
| *CCDC8* | *ITGA5* | *PRKCDBP* | *TNFAIP8L1* | *MVP* | *TNFRSF14* |
| *DBN1* | *ITPKB* | *RASIP1* | *TRPV2* | *PLK3* | *VEGFB* |
| *DCTN2* | *KRT18* | *ROBO4* | *VPS51* |  |  |
| *FAM50B* | *LAMA5* | *SELENON* | *ZNF467* |  |  |
| *FCGRT* | *MID1IP1* | *SIGIRR* |  |  |  |
| *FHL3* | *NES* | *SLC16A5* |  |  |  |
| *FNDC4* | *NOTCH1* | *SLC22A23* |  |  |  |

**Supplementary Table 4: List of canonical HIF-2α target genes defined by IPA, referring to Supplementary Fig. 1c.**

| **Canonical HIF-2α target genes defined by IPA** | | | | | | | |
| --- | --- | --- | --- | --- | --- | --- | --- |
| *ABCF2* | *C1QA* | *CLK3* | *FASN* | *INHBB* | *MYH4* | *SFTPB* | *TAF9B* |
| *ABI1* | *CA9* | *CNKSR2* | *FBLN2* | *IRS2* | *NDRG1* | *SFTPD* | *TEK* |
| *ACACA* | *CAT* | *CORO1A* | *FHL1* | *ITGAV* | *NEAT1* | *SLC11A2* | *TGFA* |
| *ACP5* | *CAV1* | *CTGF* | *FLT1* | *ITGB3* | *NEK8* | *SLC29A1* | *TMEM45A* |
| *ADM* | *CCR2* | *CYBRD1* | *FOS* | *ITIH5* | *NOS3* | *SLC2A1* | *UGP2* |
| *AKAP12* | *CCR5* | *CYP51A1* | *FXN* | *ITPR1* | *NOTCH1* | *SLC6A8* | *VEGFA* |
| *ALDOC* | *CDC42EP5* | *DTX1* | *GALR2* | *KDM3A* | *NRARP* | *SLC7A5* | *WISP2* |
| *ANGPTL4* | *CDCP1* | *EDN1* | *GJA1* | *KDM4B* | *PDPK1* | *SOD1* | *WNT1* |
| *APLN* | *CEMIP* | *EGFR* | *GLS* | *KDR* | *PFKFB3* | *SOD2* | *WNT10B* |
| *AREG* | *CHKA* | *EGLN3* | *GPX1* | *L1CAM* | *POTEG* | *SOX15* |  |
| *ARG1* | *CITED2* | *EIF5A* | *GYS2* | *LDLR* | *POU5F1* | *SOX9* |  |
| *ARG2* | *CKB* | *ENO1* | *HAMP* | *LOX* | *PRKCA* | *SPACA6* |  |
| *AXL* | *CKM* | *ENO2* | *HLA-DRB3* | *LOXL2* | *RET* | *SPAG4* |  |
| *BBC3* | *CKMT1A*  *CKMT1B* | *EPAS1* | *HMGCS1* | *MB* | *SCAP* | *SPHK1* |  |
| *BCL2* | *CKMT2* | *EPO* | *HSPA4* | *MCM3AP* | *SERPINE1* | *SREBF1* |  |
| *BNIP3* | *CLDN1* | *FAM13A* | *IGFBP3* | *MIF* | *SF3A3* | *STC2* |  |

**Supplementary Table 5. List of clustered patients and clinicopathological features, referring to Fig. 1g.**

|  | **Case number** | **Grade** | **5-year overall survival** |
| --- | --- | --- | --- |
| Group1 | case_2 | 3 |  |
|  | case_4 | 2 | ● |
|  | case_29 | 3 |  |
|  | case_37 | 3 |  |
|  | case_38 | 2 |  |
|  | case_50 | 1 | ● |
| Group2 | case_11 | 2 | ● |
|  | case_14 | 2 | ● |
|  | case_15 | 3 |  |
|  | case_20 | 2 | ● |
|  | case_26 | 2 | ● |
|  | case_28 | 2 | ● |
|  | case_34 | 2 | ● |
|  | case_55 | 2 | ● |
|  | case_63 | 2 | ● |

**Supplementary Table 6. Clinical features of chondrosarcoma patients according to *HIF1A* or *EPAS1* amplification status.**

|  | *HIF1A* loci amplification | |  |
| --- | --- | --- | --- |
| Variables | Negative (%) | Positive (%) | *P*-value |
| Dedifferentiation |  |  | 0.082 |
| + | 3 (5.45) | 2 (22.22) |  |
| – | 52 (94.55) | 7 (77.78) |  |
| Recurrence |  |  | 0.221 |
| + | 17 (30.91) | 1 (11.11) |  |
| – | 38 (69.09) | 8 (88.89) |  |
| Metastasis |  |  | 0.465 |
| + | 19 (34.55) | 2 (22.22) |  |
| – | 36 (65.45) | 7 (77.78) |  |
|  |  |  |  |
|  | *EPAS1* loci amplification | |  |
| Variables | Negative (%) | Positive (%) | *P*-value |
| Dedifferentiation |  |  | 0.041 |
| + | 1 (2.50) | 4 (16.67) |  |
| – | 39 (97.57) | 20 (83.33) |  |
| Recurrence |  |  | 0.062 |
| + | 8 (20) | 10 (41.67) |  |
| – | 32 (80) | 14 (58.33) |  |
| Metastasis |  |  | 0.243 |
| + | 11 (27.50) | 10 (41.67) |  |
| – | 29 (72.50) | 14 (58.33) |  |

Contingency tables were analyzed using a chi-square test.

**Supplementary Table 7:** **Gene list of the *Cancer stem cell* gene set, referring to Fig. 4a and Supplementary Fig. 5a.**

| **Cancer stem cell gene set** | | | | | | | |
| --- | --- | --- | --- | --- | --- | --- | --- |
| *ATG7* | *COL1A1* | *CTSB* | *GREM1* | *IGHG4* | *MAPK1* | *NOTCH3* | *PTK2* |
| *BMP7* | *COL1A2* | *DNMT1* | *HNF4A* | *IGL* | *MAPK11* | *NRG1* | *PTK2B* |
| *BRCA1* | *COL2A1* | *EGF* | *IFNG* | *JAG1* | *MAPK12* | *NRP1* | *SQSTM1* |
| *CEACAM1* | *COL3A1* | *EGFR* | *IGH* | *KDM2B* | *MAPK13* | *PIN1* | *STAT3* |
| *COL10A1* | *COL5A3* | *ERBB2* | *IGHG1* | *KDR* | *MAPK14* | *PPM1D* | *VEGFA* |
| *COL11A2* | *CSK* | *FGFR2* | *IGHG2* | *KITLG* | *MATK* | *PRNP* |  |
| *COL18A1* | *CTNNB1* | *GLDC* | *IGHG3* | *LIN28B* | *NOS2* | *PTEN* |  |

**Supplementary Table 8: Gene list of the *Multicancer invasiveness signature* gene set, referring to Fig. 4f and Supplementary Fig. 5d.**

| **Multicancer invasiveness signature gene set** | | | | | | | |
| --- | --- | --- | --- | --- | --- | --- | --- |
| *ACTA2* | *COL10A1* | *COL6A3* | *FAP* | *LOX* | *NID2* | *POSTN* | *SPOCK1* |
| *ADAM12* | *COL11A1* | *COMP* | *FBN1* | *LOXL2* | *NOX4* | *PRRX1* | *SULF1* |
| *AEBP1* | *COL1A1* | *COPZ2* | *FN1* | *LRRC15* | *NTM* | *RAB31* | *THBS2* |
| *ASPN* | *COL1A2* | *CRISPLD2* | *GLT8D2* | *LUM* | *NUAK1* | *RCN3* | *THY1* |
| *BGN* | *COL3A1* | *CTSK* | *GREM1* | *MFAP5* | *OLFML2B* | *SERPINF1* | *TIMP3* |
| *C1QTNF3* | *COL5A1* | *DCN* | *INHBA* | *MMP11* | *PCOLCE* | *SFRP4* | *TMEM158* |
| *C7orf10* | *COL5A2* | *EDNRA* | *ITGBL1* | *MMP2* | *PDGFRB* | *SNAI2* | *TNFAIP6* |
| *CDH11* | *COL6A2* | *EPYC* | *LGALS1* | *MXRA5* | *PLAU* | *SPARC* | *VCAN* |

**Supplementary Table 9: Gene list of the *Cell migration* gene set, referring to Supplementary Fig. 5d.**

| **Cell migration gene set** | | | | | | | |
| --- | --- | --- | --- | --- | --- | --- | --- |
| *ABI2* | *CALCA* | *DOCK2* | *IL12B* | *NEXN* | *PARP9* | *SEMA3B* | *TDGF1* |
| *ABI3* | *CCDC88A* | *DPYSL5* | *IL8* | *NF1* | *PF4* | *SEMA4F* | *TGFB2* |
| *ACVRL1* | *CD24* | *EGFR* | *ITGB1* | *NF2* | *PLG* | *SFTPD* | *THBS4* |
| *AIMP1* | *CD2AP* | *ENPEP* | *ITGB1BP1* | *NRD1* | *PPAP2A* | *SHH* | *THY1* |
| *ALOX15B* | *CD34* | *FEZ1* | *ITGB2* | *NRP1* | *PPAP2B* | *SHROOM2* | *TNFSF12* |
| *AMOT* | *CDH13* | *FEZ2* | *KAL1* | *NRP2* | *PRSS3* | *SIAH1* | *TNN* |
| *ANG* | *CDK5R1* | *GDNF* | *KRT2* | *NRTN* | *PTEN* | *SLIT1* | *TRIP6* |
| *ANGPTL3* | *CKLF* | *GLI2* | *LAMB1* | *NRXN1* | *RTN4* | *SLIT2* | *UBB* |
| *ARAP3* | *CLIC4* | *GTPBP4* | *LAMC1* | *NRXN3* | *S100A2* | *SPHK1* | *UNC5C* |
| *AZU1* | *CNTN4* | *HMGCR* | *MDGA1* | *OPHN1* | *S100P* | *SPON2* | *VCL* |
| *BCAR1* | *CX3CL1* | *IL10* | *MIA3* | *OTX2* | *SAA1* | *SYK* | *VEGFC* |
| *BMP10* | *CXCR2* | *IL12A* | *MYH9* | *PARD6B* | *SCG2* | *TBX5* | *WNT1* |

**Supplementary Table 10: Gene list of the *Apoptosis* gene set, referring to Fig. 6a and Supplementary Fig. 8h.**

| **Apoptosis gene set** | | | | | | | |
| --- | --- | --- | --- | --- | --- | --- | --- |
| *ADD1* | *BRCA1* | *CDK2* | *EMP1* | *GSTM1* | *LEF1* | *PRF1* | *TGFB2* |
| *AIFM3* | *BTG2* | *CDKN1A* | *ENO2* | *GUCY2D* | *LGALS3* | *PSEN1* | *TGFBR3* |
| *ANKH* | *BTG3* | *CDKN1B* | *ERBB2* | *H1F0* | *LMNA* | *PSEN2* | *TIMP1* |
| *ANXA1* | *CASP1* | *CFLAR* | *ERBB3* | *HGF* | *LPPR4* | *PTK2* | *TIMP2* |
| *APP* | *CASP2* | *CLU* | *EREG* | *HMGB2* | *LUM* | *RARA* | *TIMP3* |
| *ATF3* | *CASP3* | *CREBBP* | *ETF1* | *HMOX1* | *MADD* | *RELA* | *TNF* |
| *AVPR1A* | *CASP4* | *CTH* | *F2* | *HSPB1* | *MCL1* | *RETSAT* | *TNFRSF12A* |
| *BAX* | *CASP6* | *CTNNB1* | *F2R* | *IER3* | *MGMT* | *RHOB* | *TNFSF10* |
| *BCAP31* | *CASP7* | *CYLD* | *FAS* | *IFITM3* | *MMP2* | *RHOT2* | *TOP2A* |
| *BCL10* | *CASP8* | *DAP* | *FASLG* | *IFNB1* | *NEDD9* | *RNASEL* | *TSPO* |
| *BCL2L1* | *CASP9* | *DAP3* | *FDXR* | *IFNGR1* | *NEFH* | *ROCK1* | *TXNIP* |
| *BCL2L10* | *CAV1* | *DCN* | *FEZ1* | *IGF2R* | *PAK1* | *SAT1* | *VDAC2* |
| *BCL2L11* | *CCNA1* | *DDIT3* | *GADD45A* | *IGFBP6* | *PDCD4* | *SATB1* | *WEE1* |
| *BCL2L2* | *CCND1* | *DFFA* | *GADD45B* | *IL18* | *PDGFRB* | *SC5DL* | *XIAP* |
| *BGN* | *CCND2* | *DIABLO* | *GCH1* | *IL1A* | *PEA15* | *SLC20A1* |  |
| *BID* | *CD14* | *DNAJA1* | *GNA15* | *IL1B* | *PLAT* | *SMAD7* |  |
| *BIK* | *CD2* | *DNAJC3* | *GPX1* | *IL6* | *PLCB2* | *SOD1* |  |
| *BIRC3* | *CD38* | *DNM1L* | *GPX3* | *IRF1* | *PMAIP1* | *SOD2* |  |
| *BMF* | *CD44* | *DPYD* | *GPX4* | *ISG20* | *PPP2R5B* | *SPTAN1* |  |
| *BMP2* | *CD69* | *EBP* | *GSN* | *JUN* | *PPP3R1* | *SQSTM1* |  |
| *BNIP3L* | *CDC25B* | *EGR3* | *GSR* | *KRT18* | *PPT1* | *TAP1* |  |

**Supplementary Table 11: Gene list of the *P53 pathway* gene set, referring to Fig. 6b and Supplementary Fig. 8h.**

| **P53 pathway gene set** | | | | | | | |
| --- | --- | --- | --- | --- | --- | --- | --- |
| *ABAT* | *CCP110* | *EPHA2* | *HMOX1* | *MXD1* | *PRMT2* | *SAT1* | *TNFSF9* |
| *ABCC5* | *CD81* | *EPHX1* | *HRAS* | *MXD4* | *PROCR* | *SDC1* | *TNNI1* |
| *ABHD4* | *CD82* | *EPS8L2* | *HSPA4L* | *NDRG1* | *PTPN14* | *SEC61A1* | *TOB1* |
| *ACVR1B* | *CDH13* | *ERCC5* | *IER3* | *NHLH2* | *PTPRE* | *SERPINB5* | *TP53* |
| *ADA* | *CDK5R1* | *F2R* | *IER5* | *NINJ1* | *PVT1* | *SERTAD3* | *TP63* |
| *ADCK3* | *CDKN1A* | *FAM162A* | *IFI30* | *NOL8* | *RAB40C* | *SESN1* | *TPD52L1* |
| *AEN* | *CDKN2A* | *FAS* | *IKBKAP* | *NOTCH1* | *RAD51C* | *SFN* | *TPRKB* |
| *AK1* | *CDKN2AIP* | *FBXW7* | *IL1A* | *NUDT15* | *RAD9A* | *SLC19A2* | *TRAF4* |
| *ALOX15B* | *CDKN2B* | *FDXR* | *INHBB* | *NUPR1* | *RALGDS* | *SLC35D1* | *TRAFD1* |
| *ANKRA2* | *CEBPA* | *FGF13* | *IP6K2* | *OSGIN1* | *RAP2B* | *SLC3A2* | *TRIAP1* |
| *APAF1* | *CGRRF1* | *FOS* | *IRAK1* | *PCNA* | *RB1* | *SLC7A11* | *TRIB3* |
| *APP* | *CLCA2* | *FOXO3* | *ISCU* | *PDGFA* | *RCHY1* | *SOCS1* | *TSC22D1* |
| *ATF3* | *CSRNP2* | *FUCA1* | *ITGB4* | *PERP* | *RETSAT* | *SP1* | *TSPYL2* |
| *BAIAP2* | *CTSD* | *GADD45A* | *JAG2* | *PHLDA3* | *RGS16* | *SPHK1* | *TXNIP* |
| *BAK1* | *CTSF* | *GLS2* | *JUN* | *PIDD* | *RHBDF2* | *ST14* | *UPP1* |
| *BAX* | *CYFIP2* | *GM2A* | *KIF13B* | *PITPNC1* | *RNF19B* | *STEAP3* | *VAMP8* |
| *BLCAP* | *DCXR* | *GNB2L1* | *KLF4* | *PLK2* | *RPL18* | *STOM* | *VDR* |
| *BMP2* | *DDB2* | *GPX2* | *KLK8* | *PLK3* | *RPL36* | *TAP1* | *VWA5A* |
| *BTG1* | *DDIT3* | *H2AFJ* | *KRT17* | *PLXNB2* | *RPS12* | *TAX1BP3* | *WRAP73* |
| *BTG2* | *DDIT4* | *HBEGF* | *LDHB* | *PMM1* | *RPS27L* | *TCHH* | *WWP1* |
| *CASP1* | *DEF6* | *HDAC3* | *LIF* | *POLH* | *RRAD* | *TCN2* | *XPC* |
| *CCND2* | *DGKA* | *HEXIM1* | *LRMP* | *POM121* | *RRP8* | *TGFA* | *ZBTB16* |
| *CCND3* | *DNTTIP2* | *HINT1* | *MAPKAPK3* | *PPM1D* | *RXRA* | *TGFB1* | *ZFP36L1* |
| *CCNG1* | *DRAM1* | *HIST1H1C* | *MDM2* | *PPP1R15A* | *S100A10* | *TM4SF1* | *ZMAT3* |
| *CCNK* | *EI24* | *HIST3H2A* | *MKNK2* | *PRKAB1* | *S100A4* | *TM7SF3* | *ZNF365* |

**Supplementary Table 12: List of oligomer sequences used in this study.**

| **siRNA sequences** | |
| --- | --- |
| Name | Sequence (5′→3′) |
| *HIF1A* siRNA - sense | GUGGUUGGAUCUAACACUA(dTdT) |
| *HIF1A* siRNA - antisense | UAGUGUUAGAUCCAACCAC(dTdT) |
| *EPAS1* siRNA - sense | ACUACGUCCUGAGUGAGAU(dTdT) |
| *EPAS1* siRNA - antisense | AUCUCACUCAGGACGUAGU(dTdT) |
| *IDH1* siRNA - sense | CUUGCUGAAUGUUUCCAAU(dTdT) |
| *IDH1* siRNA - antisense | AUUGGAAACAUUCAGCAAG(dTdT) |
| *IDH2* siRNA - sense | CUGUACAUGAGCACCAAGA(dTdT) |
| *IDH2* siRNA - antisense | UCUUGGUGCUCAUGUACAG(dTdT) |
|  | |
| **Sanger sequencing related primers** | |
| Name | Sequence(5′→3′) |
| *IDH1* gDNA-F | AATGAGCTCTATATGCCATCACTG |
| *IDH1* gDNA-R | TTCATACCTTGCTTAATGGGTGT |
| *IDH2* gDNA-F | GGGGTTCAAATTCTGGTTGA |
| *IDH2* gDNA-R | CTAGGCGAGGAGCTCCAGT |
| Abbreviations: gDNA, genomic DNA ;F, forward; R, reverse | |
|  |  |
| **shRNA cloning primers** | |
| Name | Sequence(5′→3′) |
| scramble shRNA pLKO.1-F (control) | CCGGAAACAAGATGAAGAGCACCAACTCGA  GTTGGTGCTCTTCATCTTGTTTTTTTTG |
| scramble shRNA pLKO.1-R (control) | AATTCAAAAAAAACAAGATGAAGAGCACCA  ACTCGAGTTGGTGCTCTTCATCTTGTTT |
| *EPAS1* shRNA oligo pLKO.1-F | CCGGCAGTACCCAGACGGATTTCAACTCGA  GTTGAAATCCGTCTGGGTACTGTTTTTG |
| *EPAS1* shRNA oligo pLKO.1-R | AATTCAAAAACAGTACCCAGACGGATTTCA  ACTCGAGTTGAAATCCGTCTGGGTACTG |
| Abbreviations: F, forward; R, reverse |  |
| **CRISPR related primers** | |
| Name | Sequence(5′→3′) |
| *EPAS1*_guideRNA214rvU6senselentiCrispr | CACCGAGGCTGTCAGACCCGAAAAG |
| *EPAS1*_guideRNA214rvU6antisenselentiCrispr | AAACCTTTTCGGGTCTGACAGCCTC |
| *EPAS1*_OntargetGuideRna214rv Left for T7E1 assay | CGTACAATCCTCGGCAGTGT |
| *EPAS1*_OntargetGuideRna214rv Right for T7E1 assay | GGGGTGGAGAAAGGTGACTG |
