# Supplementary material for: A system-level approach identifies HIF-2α as a critical regulator of chondrosarcoma progression
Source: Nat Commun. 2020 Oct 6;11:5023. doi: 10.1038/s41467-020-18817-7 (PMC7538956; doi:10.1038/s41467-020-18817-7)
Supplement: Supplementary file 2 — Description of Additional Supplementary Files [file 41467_2020_18817_MOESM2_ESM.pdf]

Description of Additional Supplementary Files

File Name: Supplementary Data 1
Description: Gene list of six gene modules identified by WGCNA, referring to Fig. 1a and Supplementary Fig. 1a.

File Name: Supplementary Data 2
Description: Patient information for 65 chondrosarcoma specimens, referring to Fig. 1e, f and Supplementary Fig. 5h, i.

File Name: Supplementary Data 3
Description: List of downregulated genes following HIF-1α or HIF-2α knockdown in SW1353 cells, referring to Fig. 3a.

File Name: Supplementary Data 4
Description: List of PCR primers.
