# Supplementary material for: A system-level approach identifies HIF-2α as a critical regulator of chondrosarcoma progression
Source: Nat Commun. 2020 Oct 6;11:5023. doi: 10.1038/s41467-020-18817-7 (PMC7538956; doi:10.1038/s41467-020-18817-7)
Supplement: Supplementary file 7 — Reporting Summary [file 41467_2020_18817_MOESM7_ESM.pdf]

nature research

Corresponding author(s): Jin-Hong Kim

Last updated by author(s): Sep 7, 2020

Reporting Summary

Nature Research wishes to improve the reproducibility of the work that we publish. This form provides structure for consistency and transparency in reporting. For further information on Nature Research policies, see our Editorial Policies and the Editorial Policy Checklist.

Statistics

For all statistical analyses, confirm that the following items are present in the figure legend, table legend, main text, or Methods section.

n/a	Confirmed	
☐	☒	The exact sample size (*n*) for each experimental group/condition, given as a discrete number and unit of measurement
☐	☒	A statement on whether measurements were taken from distinct samples or whether the same sample was measured repeatedly
☐	☒	The statistical test(s) used AND whether they are one- or two-sided<br>*Only common tests should be described solely by name; describe more complex techniques in the Methods section.*
☒	☐	A description of all covariates tested
☐	☒	A description of any assumptions or corrections, such as tests of normality and adjustment for multiple comparisons
☐	☒	A full description of the statistical parameters including central tendency (e.g. means) or other basic estimates (e.g. regression coefficient) AND variation (e.g. standard deviation) or associated estimates of uncertainty (e.g. confidence intervals)
☐	☒	For null hypothesis testing, the test statistic (e.g. *F*, *t*, *r*) with confidence intervals, effect sizes, degrees of freedom and *P* value noted<br>*Give P values as exact values whenever suitable.*
☒	☐	For Bayesian analysis, information on the choice of priors and Markov chain Monte Carlo settings
☐	☒	For hierarchical and complex designs, identification of the appropriate level for tests and full reporting of outcomes
☐	☒	Estimates of effect sizes (e.g. Cohen's *d*, Pearson's *r*), indicating how they were calculated

Our web collection on statistics for biologists contains articles on many of the points above.

Software and code

Policy information about availability of computer code

Data collection	Histological. immunohistochemistry staining images, underside of transwell images were acquired with the DS-Ri2 camera (Nikon). For immunofluorescence detection, the immunostained tissues were imaged with a laser scanning confocal microscope (Carl Zeiss, LSM700). Array data was obtained using the GeneChip® Human Gene 2.0 ST Array (Affymetrix). Images of sphere formation assay were obtained with Axio Observer Z1 (Zeiss). The wound area were captured by a digital camera under a microscope (EVOS XL Core Cell Imaging System, Thermo Fisher Scientific). For gelatin degradation assay and BrdU assay, images were obtained using a fluorescence microscope (EEVOS FL Cell Imaging System, Thermo Fisher Scientific). For MMP activity assay, fluorescence intensity was detected using a Spectramax Gemini microplate fluorescence reader (Molecular Device). Colorimetric assay was obtained from Varioskan LUX. qRT-PCR was performed with a StepOnePlus Real-Time PCR System (Applied Biosystems). Flow cytometry analysis was performed on a FACS Canto II flow cytometer (BD Biosciences). Immunoblot images were obtained using iBright FL1000 (Thermo Fisher Scientific)
Data analysis	Affymetrix® GeneChip Command Console® (AGCC) ver. 1.32.0, STAR ver. ver. 0.3-7, HOMER ver. 1.2.2, WGCNA ver. 1.69, prcomp ver. 0.1-0, GLAD ver. 2.36, k -means clustering ver. 1.0 R packages were used in this study. R program ver. 3.4.3 was used. IBM SPSS ver. 22 statistical software, GraphPad Prism ver. 8, XLSTAT ver. 2018. 7, IPA ver. 8.7, Cytoscape software ver. 3.6.1, VisANT ver. 5.51, SnapGene Viewer 4.2.6, Enrichr ver. June. 18th 2018, Compusyn software version ver. 1.0, MSigDB ver. 6.0, and GSEA ver. 3.0 were used in this study. ImageJ ver. 1.52P and Image-Pro Premier ver. 9.2 were used for image data quantification in this study. CRISPOR search algorithm (https://crispor.tefor.net/crispor.py; ver. 4.98) was used in this study. BD FACSDiva software ver. 8.0.1 was used in this study.

For manuscripts utilizing custom algorithms or software that are central to the research but not yet described in published literature, software must be made available to editors and reviewers. We strongly encourage code deposition in a community repository (e.g. GitHub). See the Nature Research guidelines for submitting code & software for further information.

Data

Policy information about availability of data

All manuscripts must include a data availability statement. This statement should provide the following information, where applicable:

- Accession codes, unique identifiers, or web links for publicly available datasets
- A list of figures that have associated raw data
- A description of any restrictions on data availability

The expression profiling and genome variation profiling data referenced during the study are available in a public repository from the NCBI (https://www.ncbi.nlm.nih.gov) websites. For the transcriptome analysis, the following datasets were used: chondrosarcoma patient dataset (GSE12475), murine chondrocyte dataset (GSE73659), and the primary cultured chondrosarcoma cells dataset (GSE47823). For the copy number alteration analysis, chondrosarcoma patient dataset (GSE12532) was used. HIF-2α ChIP-Seq datasets (GSM3417828, GSM3417842) were used for the ChIP-Seq analysis. The original transcriptome datasets that were produced in this study are deposited in the Gene Expression Omnibus (GSE156565). Cancer stem cell gene set was obtained from IPA. Multicancer invasiveness signature, Cell migration, Apoptosis, and P53 Pathway gene sets were obtained from MSigDB v.6.0 (https://software.broadinstitute.org/gsea/msigdb/index.jsp).

Field-specific reporting

Please select the one below that is the best fit for your research. If you are not sure, read the appropriate sections before making your selection.

- [x] Life sciences
- [ ] Behavioural & social sciences
- [ ] Ecological, evolutionary & environmental sciences

For a reference copy of the document with all sections, see nature.com/documents/nr-reporting-summary-flat.pdf

Life sciences study design

All studies must disclose on these points even when the disclosure is negative.

Sample size	We determined sample sizes based on our previous experiences: Kim et al., 2014, Cell, 156(4), 730-43; Kang et al., 2019, Sci. Transl. Med., 11 (486), eaar6659; Kim et al., 2019, Nat. Commun., 10(1), 4898. All experiments were conducted on at least three independent biological replicates. This is based on the number of biological replicates required for statistical analyses. For each experiment, sample size and the number of independent biological replicates are indicated in the corresponding figure legends.
Data exclusions	No data were excluded from the analyses.
Replication	All attempts at replication were successful.
Randomization	For in vivo experiments, female athymic nude mice (BALB/c nu/nu) were randomly assigned to each experimental group with approximately equal numbers in each group.
Blinding	Investigators were blinded during data collection. All histological and immunohistochemical specimens were evaluated independently by two oncologists with specific expertise in human sarcomas. The oncologists were blinded to the labeling of the specimens during data analysis.

Reporting for specific materials, systems and methods

We require information from authors about some types of materials, experimental systems and methods used in many studies. Here, indicate whether each material, system or method listed is relevant to your study. If you are not sure if a list item applies to your research, read the appropriate section before selecting a response.

Materials & experimental systems

n/a	Involved in the study	
[ ]	[x]	Antibodies
[ ]	[x]	Eukaryotic cell lines
[x]	[ ]	Palaeontology and archaeology
[ ]	[x]	Animals and other organisms
[ ]	[x]	Human research participants
[x]	[ ]	Clinical data
[x]	[ ]	Dual use research of concern

Methods

n/a	Involved in the study	
[x]	[ ]	ChIP-seq
[ ]	[x]	Flow cytometry
[x]	[ ]	MRI-based neuroimaging

Antibodies

Antibodies used	Anti-HIF-2α antibody (cat. No. sc-13596, Santa Cruz Biotechnology; 1:200), anti-Actin antibody (cat. No. sc-1615, Santa Cruz Biotechnology; 1:2000), anti-HIF-2α antibody (cat. No. NB100-122, Novus Biologicals; 1:2000), peroxidase goat anti-mouse IgG (cat.

No. 115-035-044, Jackson ImmunoResearch Labs; 1:10000), and peroxidase goat anti-rabbit IgG (cat. No. 111-035-003, Jackson ImmunoResearch Labs; 1:10000) were used for western blot. Anti-MMP1 antibody (cat. No. sc-21731, Santa Cruz Biotechnology; 1:100), anti-human mitochondria antibody (cat. No. MAB1273, Millipore; 1:50), anti-HIF-2α antibody (cat. No. NB100-122, Novus Biologicals; 1:50), and biotin-SP-conjugated goat anti-rabbit IgG (cat. No. 711-065-152, Jackson ImmunoResearch Labs; 1:200) were used for IHC, Anti-BrdU antibody (cat. No. sc-32323, Santa Cruz Biotechnology; 1:200), anti-Ki67 antibody (cat. No. ab15580, Abcam; 1:50), anti-human mitochondria antibody (cat. No. MAB1273, Millipore; 1:50), anti-Lamin B antibody (cat. No. sc-6216, Sata Cruz Biotechnology; 1:50), anti-HIF-2α antibody (cat. No. NB100-122, Novus Biologicals; 1:50), dylight 488-conjugated anti-mouse IgG + IgM (cat. No. 315-485-044, Jackson ImmunoResearch Labs; 1:200), anti-rabbit IgG Alexa Fluor 488 (cat. No. A-21206, Thermo Fisher Scientific; 1:200), anti-rabbit IgG Alexa Fluor 647 (cat. No. A-31573, Thermo Fisher Scientific; 1:200), anti-mouse IgG Alexa Fluor 594 (cat. No. A-21203, Thermo Fisher Scientific; 1:200), and anti-goat IgG Alexa Fluor 488 (cat. No. A-11055, Thermo Fisher Scientific; 1:200) were used for IF. Normal mouse IgG (cat. No. sc-2025, Santa Cruz Biotechnology), and goat anti-mouse IgG-B (cat. No. sc-2039, Santa Cruz Biotechnology) were used for blocking and negative controls.

Validation

All purchased antibodies were validated by the manufacturer and various published data. Anti-HIF-2α antibody (cat. No. sc-13596) is recommended for detection of HIF-2α of human origin by Western blot. Anti-MMP1 antibody (cat. No. sc-21731) is recommended for detection of MMP1 of human origin by IHC. Anti-Actin antibody (cat. No. sc-1615) is recommended for detection of Actin of human origin by western blot. Anti-BrdU antibody (cat. No. sc-32323) is recommended for detection of BrdU by IF. Anti-Ki67 antibody (cat. No. ab15580) is recommended for detection of Ki67 of human origin by IF. Anti-human mitochondria antibody (cat. No. MAB1273) is recommended for detection of mitochondrial protein component of human origin by IHC. This antibody dose not cross react with mouse tissue. Anti-Lamin B antibody (cat. No. sc-6216) is recommended for detection of Lamin B of human and mouse origin is recommended by IF. Anti-HIF-2α antibody (cat. No. NB100-122) is recommended for detection of HIF-2α of human origin by IHC, IF, or western blot.

Eukaryotic cell lines

Policy information about cell lines

Cell line source(s)

Chondrosarcoma cell lines SW1353 and OUMS-27 were purchased from ATCC and JCRB Cell Bank, respectively. Chondrosarcoma cell line JJ012 was kindly provided by Professor Joel A. Block (Rush University Medical Centre, Chicago, USA). HEK293T cells derived from human embryonic kidney cells were purchased from ATCC.

Authentication

Cell lines were tested and authenticated using short-tandem repeat profiling by the Korean Cell Line Bank and CosmoGenetech Inc.

Mycoplasma contamination

Cell lines were verified to be free of mycoplasma contamination.

Commonly misidentified lines
(See ICLAC register)

No commonly misidentified cell lines were used.

Animals and other organisms

Policy information about studies involving animals; ARRIVE guidelines recommended for reporting animal research

Laboratory animals

For all xenograft models, female athymic nude mice (BALB/c nu/nu, 4 weeks old) were used. Mice were maintained in the following conditions: temperature: 23~25 ℃ , relative humidity : 45~65%, and lighting cycle : 12 h light/12 h dark cycle. The above information on laboratory animal housing conditions are described in the Method section.

Wild animals

This study did not involve wild animals.

Field-collected samples

This study did not involve samples collected from the field.

Ethics oversight

All animal studies were approved by the Seoul National University Institutional Animal Care and Use Committees (IACUC; IACUC No. SNU-151202-7-3 and SNU-151216-2-6). Animal experiments were reported in accordance with the ARRIVE guidelines (https://www.nc3rs.org.uk/arrive-guidelines).

Note that full information on the approval of the study protocol must also be provided in the manuscript.

Human research participants

Policy information about studies involving human research participants

Population characteristics

Chondrosarcoma, enchondroma, osteochondroma, and normal cartilage specimens were used in this study. Both male and female were included in this study. Each chondrosarcoma biopsy was individually examined and graded by the state-certified pathologists at US Biomax according to the WHO guidelines.

Recruitment

Human specimens were obtained from the National Cancer Institute Cooperative Human Tissue Network (CHTN). Written informed consents were obtained from all participants by the CHTN. Tissue arrays of various cartilage tumors (T261a) and normal cartilage and chondrosarcoma (OS805) were obtained from the US Biomax, Inc.

Ethics oversight

Before obtaining biopsies, the study was approved by the Institutional Review Board (IRB) of the Seoul National University (IRB No. E1611/003-008 and E1803/003-007).

Note that full information on the approval of the study protocol must also be provided in the manuscript.

Flow Cytometry

Plots

Confirm that:

- [x] The axis labels state the marker and fluorochrome used (e.g. CD4-FITC).
- [x] The axis scales are clearly visible. Include numbers along axes only for bottom left plot of group (a 'group' is an analysis of identical markers).
- [x] All plots are contour plots with outliers or pseudocolor plots.
- [x] A numerical value for number of cells or percentage (with statistics) is provided.

Methodology

Sample preparation	For the analysis of apoptosis, SW1353 cells were pre-treated with 10 µM TC-S7009 or DMSO (vehicle) for 48 h and additionally treated with 5 µg/ml cisplatin or double distilled water (vehicle) for 24 h. Subsequently, the cells were stained with the FITC-Annexin V Apoptosis Detection Kit (Sigma Aldrich, APOAF) according to the manufacturer's protocol.
Instrument	FACS Canto II flow cytometer
Software	BD FACSDivaTM Software was used to analyze the data.
Cell population abundance	At least 1 million cells were analyzed by flow cytometry.
Gating strategy	Cells were gated by forward scatter (FSC) and side scatter (SSC) area (A) according to cell size and granularity to remove debris and large clumps. Singlet cells were then selected using FSC-A and FSC-height (H).

- [x] Tick this box to confirm that a figure exemplifying the gating strategy is provided in the Supplementary Information.
